# Structural and functional role of disulphide bonds and substrate binding residues of the human beta-galactoside alpha-2,3-sialyltransferase 1 (hST3Gal1)

**DOI:** 10.1038/s41598-019-54384-8

**Published:** 2019-11-29

**Authors:** Maria Elena Ortiz-Soto, Sabine Reising, Andreas Schlosser, Jürgen Seibel

**Affiliations:** 1 Institut für Organische Chemie, Universität Würzburg, Am Hubland, 97074 Würzburg, Germany; 2 Rudolf-Virchow-Zentrum für Experimentelle Biomedizin, Universität Würzburg, Josef-Schneider Str. 2, Haus D15, 97080 Würzburg, Germany

**Keywords:** Transferases, Glycobiology

## Abstract

Overexpression of hST3Gal1 leads to hypersialylation of cell-surface glycoconjugates, a cancer-associated condition that promotes cell growth, migration and invasion. Upregulation of this enzyme in ovarian cancer is linked to cancer progression and metastasis, contributing also to chemotherapy resistance. Strategies for preventing metastasis include the inhibition of hST3Gal1, which demands structure-based studies on its strict regioselectivity and substrate/donor preference. Herein we describe the contribution of various residues constituting donor CMP-Neu5Ac and acceptor Galβ1-3GalNAc-R binding sites to catalysis. Removal of hydrogen bonds and/or stacking interactions among substrates and residues Y191, Y230, N147, S148 and N170 affected the enzyme’s activity to a different extent, revealing the fine control needed for an optimal catalytic performance. To gain further understanding of the correlation among structure, activity and stability, the *in vitro* role of hST3Gal1 disulphide bonds was analysed. As expected, disruption of the Glycosyltransferase family 29 (GT29) invariant bond C142-C281, as well as the ST3Gal1 subfamily conserved disulphide C61-C139 inactivates the enzyme. While disulphide C59-C64 is not essential for function, its absence reduces the activity (k_cat_) for donor and acceptor substrates to about 67 and 72%, respectively, and diminishes the enzyme’s melting temperature (T_m_) by 7 °C.

## Introduction

Glycans of different complexity that decorate the cell surface of organisms of the three domains of life are involved in a variety of physiological and pathological events^1–3^. Glycoconjugates containing terminal sialic acids participate in biological recognition processes such as system embryogenesis, cell trafficking, transmembrane signalling, immunological regulation, tumor invasion and host-pathogen interactions^2,4–6^. In mammals the transfer of sialic acid from donor CMP-Sia is catalysed by twenty sialyltransferases classified into the Glycosyltransferase family 29 (GT29) (http://www.cazy.org/)^7,8^. GT29 enzymes are Type II membrane proteins confined to the Golgi apparatus. They present a transmembrane domain (16–20 amino acids) for Golgi retention, which is flanked by a short N-terminal cytoplasmic tail and a C-terminal stem region followed by the catalytic domain^9^. Sialyltransferases vary in terms of their substrate specificity, tissue distribution, and biochemical parameters^8^. They are classified into subfamilies ST3Gal1-6, ST6Gal1-2, ST6GalNAc1-6 and ST8Sia1-6, based on their regioselectivity for the transferred sialic acid and their acceptor specificity^10,11^. ST3Gal and ST6Gal enzymes form α2-3- and α2-6-linkages, respectively, on oligosaccharide chains containing a terminal galactose. ST6GalNAc and ST8Sia generate α2-6- and α2-8-bonds, respectively. Proteins in the former subfamily transfer sialic acid onto terminal GalNAc residues and enzymes from the latter recognise acceptors carrying an external sialic acid unit. Sialyltransferases are inverting enzymes regarding the configuration of the anomeric centre of sialic acid upon transfer, which occurs via a direct displacement S_N_2-like mechanism^12,13^. Members within each subfamily show a typical pattern of conserved cysteine residues involved in the formation of disulphide bonds that are important for proper folding and activity^9,14^. Multiple sequence alignments of the catalytic domain reveal highly conserved regions denoted as sialylmotifs L (long), S (short), III and VS (very short). Mutational studies performed during the last decades have contributed to a better understanding of the role of residues within the sialylmotifs^12,15,16^. Residues contributing to substrate binding are localized in the sialylmotifs L and S, while amino acids from sialylmotifs III and VS participate in catalytic activity^13,17^. Crystal structures of sialyltransferases from the four subfamilies exhibit a single Rossmann-like (GT-A variant 2) fold consisting of seven central β-strands flanked by multiple α-helices^13,15,17–19^. Differences in the helical and loop segments that include various insertion and deletion sequences are presumably responsible for the rigorous linkage and/or acceptor specificities displayed by sialyltransferases from family GT29.

Even minor changes in glycosylation provoke anomalous cellular processes. Hypersialylation for instance correlates with cancer stage and disease prognosis^20,21^. High expression levels of hST3Gal1 and other sialyltransferases are observed in different cancer types; increased α-2,3 sialylated core 1 O-glycans are common in breast, bladder and colon cancer^20^. Overexpression of hST3Gal1 is also observed in ovarian, prostatic and pancreatic cancer^8^. Sialyltransferases and other enzymes that participate in the synthesis and processing of sialic acids are therefore considered as potential targets for drug development, and inhibition of sialyltransferases has been proposed as a strategy to prevent metastasis of different types of cancer^8^. The elucidation of protein-ligand molecular interactions with donor and acceptor molecules are significant components of inhibitor design. Although the three-dimensional structure of hST3Gal1 has not been solved, the structure of its closely related porcine homolog ST3Gal1 (pST3Gal1) to which it shares 85% identity was determined in the apo-form, as a binary complex with acceptor Galβ1,3GalNAcα-PhNO_2_ and as a ternary complex with the acceptor and CMP^13^. The latter was used as a template for modelling hST3Gal1 and to investigate the role of various residues in the donor and acceptor binding sites that were not previously explored. The effect of altering residues N147, S148 and N170 which are located at the donor binding site, and residue V315, which is adjacent to the catalytic H316 is described. The contribution of residues Y191 and Y230 to catalysis was also determined and compared to corresponding mutations reported in pST3Gal1^12^. ST3Gal1 enzymes possess three disulphide bonds, with the pair formed by C142 and C281 being completely conserved among all members of family GT29. In the present work we explored the implication of disulphide bonds in activity and protein stability. While native disulphides C61-C139 and C142-C281 are necessary for activity, the bond formed between C59 and C64 is not absolutely required for proper folding and activity, yet it contributes to the enzyme thermal stability in *in vitro* assays.

## Results and Discussion

### Homology modelling of hST3Gal1 and selection of variants in donor and acceptor binding sites

Two homology models of hST3Gal1 based on the apo- and substrate-bound crystal structures of pST3Gal1 (PDB codes 2wml and 2wnb, respectively)^13^ were generated employing the SWISS-MODEL server^22^. Structural alignment of models and templates performed with UCSF Chimera (http://www.rbvi.ucsf.edu/chimera) provided global RMSD (root-mean-square deviation) values of 0.073 and 0.063 Å for the models based on the apo- and substrate-bound structures, respectively.

**Figure 1:**
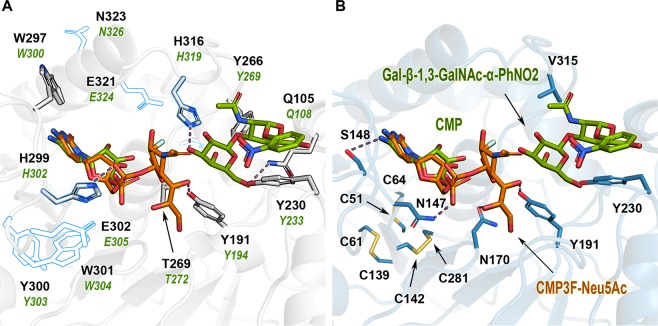
A view of the active site of human and porcine ST3Gal1 enzymes. (**A**) Cartoon representation of pST3Gal1 (PDB 2wnb). Positions previously mutated in pST3Gal1^12^ and hST3Gal1^23^ are shown as white sticks and as blue outlines, respectively. In the latter enzyme, only H316 and H299 are part of the active/binding site. Amino acid sequence numbering for porcine and human enzymes is in green and black font, respectively. (**B**) Cartoon representation of modelled hST3Gal1. Positions selected for mutagenesis of hST3Gal1 in this paper are displayed as blue sticks. CMP and acceptor Gal-β-1,3-GalNAc-α-PhNO_2_ from pST3Gal1 (PDB 2wnb, green sticks) as well as CMP3F-Neu5Ac from the sialyltransferase CstII of *Campylobacter jejuni* (PDB 1ro7, orange sticks) are shown in the active site. Possible interactions between binding site residues and substrates (<4 Å) are displayed as dashed lines. Disulphide bonds are located >16 Å away from donor and acceptor binding sites. Structures of CstII and pST3Gal1 as well as the model of hST3Gal1 were aligned with UCSF Chimera (http://www.rbvi.ucsf.edu/chimera).

As mentioned above, the structure of pST3Gal1 displays seven β-strands flanked by 12 α-helices, with the donor and acceptor binding sites localized in a cleft formed by the β-core and some α-helices and loops of variable length^13^. Residues constituting hST3Gal1 substrate binding sites show a clear spatial alignment with those in the porcine homolog in both models (Supplementary Information, Fig. S1). On this basis we assumed that changes induced in the side chain of some amino acids upon substrate binding are similar in both enzymes and hence employed the model based on pST3Gal1 bound-structure (2wnb) to identify target residues for mutagenesis (Fig. 1 and Supplementary Information, Fig. S1).

Enzymes from the four sialyltransferase subfamilies, namely ST3Gal1-6, ST6GalNAc1-6, ST8Sia1-6 and ST6Gal1-2 possess low overall sequence identity when compared with one another. They present however conserved regions known as sialylmotifs L, S, III and VS^23,24^, that include some of the residues forming the substrates binding sites and a pair of conserved cysteines. Sialylmotif L is constituted by 53 amino acids^25^, spanning in hST3Gal1-6 from the invariant C139 to Y191, while sialylmotif S includes 24 amino acids (from P267 to G290) and contains the other conserved cysteine (C281). Sialylmotif III is only 4 amino acids long (^299^H-Y-Y/f/w/h-E/D/g/q/n/s/t/k^302^) and contains an invariant histidine (H299) and a conserved tyrosine (Y300). The sialylmotif VS comprises 6 residues (^316^H-X-X-X-X-E^321^), starting with the catalytic base H316 and ending with a fully conserved glutamic acid. Sialylmotif sequences for subfamily ST3Gal1-6 are shown in Supplementary Information, Fig. S2.

Residues equivalent to Q105, Y191, Y230, Y266, T269, W297, H299, F310, R311, K312 and the catalytic base H316 were previously mutated in pST3Gal1^12^ (Fig. 1A). Likewise, various mutations were reported for hST3Gal1, albeit only variants H299A/Y and H316A were explored within the acceptor/donor binding sites^23^ (Fig. 1A). Exchange of H299, Y300 and H316 by alanine rendered hST3Gal1 variants inactive, while mutants W301F, E302Q, and E321Q retained 25–80% of the wild type activity^23^.

Residues Y191, H299 and catalytic base H316 belong to the sialylmotifs L, III and VS, respectively. Y266 precedes sialylmotif S, while T269 is part of it. Here we investigated the effect of replacing residues N147, S148, N170, Y191, Y230, and V315 on hST3Gal1 activity. All residues except the latter interact with donor and/or acceptor substrates (Fig. 1B). Residues N147, S148, N170 and Y191 belong to the sialylmotif L.

V315 is replaced by either tyrosine or tryptophan in enzymes from the subfamily ST6Gal1-2, which transfer sialic acid to the C6-hydroxyl group of the terminal galactose unit of glycoconjugates and produce α2-6-linkages. Aromatic stacking between Y369 (the residue found at the equivalent position to V315 in hST6Gal1) with the nonpolar face of the terminal galactosyl moiety of the carbohydrate acceptor possibly contributes to the regioselectivity of ST6Gal enzymes (Supplementary Information, Fig. S3). This and other interactions mostly involving tyrosine residues, place the galactose C6-hydroxyl group close to the Nε atom of the catalytic base histidine and the C2 position of Neu5Ac^15,17^. Mutation of the equivalent tyrosine residue by alanine in rat ST6Gal1 (Y366) renders the enzyme inactive^15^. We analysed the replacement of V315 by alanine as well as tyrosine and analysed the effect of the mutations on the enzyme’s activity and regioselectivity.

Another structural feature that differentiates GT29 enzymes is the subfamily-specific conservation of disulphide bonds. Only the disulphide bond formed between C142-C281 is conserved throughout the GT29 family. Enzymes from the subgroups ST3Gal-1, -2 and -6 contain 3 disulphide bonds in the catalytic domain, while ST3Gal-3 and -5 can only form two bonds.

We analysed the significance of the three disulphide bonds of hST3Gal1 in activity and stability. For this purpose, cysteine pairs involved in disulphide bonds were replaced by serine in the double variants C59S-C64S, C61S-C139S and C142S-C281S.

A 3DM superfamily database (Bio-Prodict) containing originally 3642 sequences of annotated, putative and predicted GT29 sialyltransferases from different organisms was utilized to analyse the conservation of residues involved in contacts with the donor and acceptor molecules, after eliminating redundancy and sequences with less than 300 amino acids. The 3DM superfamily comprises sequences with a common fold and uses a structural core alignment to generate a phylogenetic tree, thereby forming groups of sequences that are classified as subfamilies^26^. Each group contains sequences that are at least 80% identical at the core (aligned) positions^27^. Only 271 and 43 non-redundant sequences belonging to the subfamily ST3Gal1-6 and the subgroup ST3Gal1, respectively, were employed for analyses herein.

An N-terminally truncated version of hST3Gal1 (Δ45) lacking the transmembrane region was fused to the maltose binding protein (MBP) to improve its solubility and was co-expressed in *E.coli* Origami2 (DE3) with chaperone/foldases sulfhydryl oxidase and disulphide isomerase C, as reported previously^28^. Both chaperone/foldases assist in the proper formation of disulphide bonds^29^. Variants of hST3Gal1 were constructed using the wild-type gene cloned in the vector pMAL-C5x as a template and enzymes were expressed and purified by immobilized metal affinity chromatography.

### Circular dichroism spectroscopy and kinetic characterization of variants

The integrity of the secondary structure of MBP-fused hST3Gal1 variants was explored by circular dichroism prior to their kinetic analysis. All variants (except N170A, for which not enough protein was obtained for structural analysis), showed a similar CD spectrum to the wild-type hST3Gal1 (Supplementary Information, Fig. S4). This excludes the possibility of mutation-induced secondary structure modification. The CD spectrum of the wild-type enzyme that appears in Supplementary Information, Fig. S4 was reported earlier^28^. Because the appendage/cleavage of MBP does not modify the acceptor specificity nor the kinetic behaviour of porcine and human ST3Gal1^12,13,28^, further characterization of all variants was performed with the fusion constructs.

hST3Gal1 is an inverting enzyme that catalyses the transfer of sialic acids onto a terminal galactose in the core 1 oligosaccharide motif Gal-β-1,3-GalNAc-R of Ser/Thr O-glycans and ganglio-series glycolipids to form α2,3 linkages^30,31^. Activity and kinetic parameters of wild-type hST3Gal1 and its variants were determined via a multi-enzymatic assay based on the catalysed release of CMP from donor CMP-Neu5Ac, which is coupled to the oxidation of NADH. Gal-β-1,3-GalNAc-α-*O*-Bn was employed as acceptor of the Neu5Ac moiety. The effect of replacing selected residues on the activity of hST3Gal1 (Table 1) is discussed in later sections.

**Table 1:** Kinetic parameters of MBP-fused hST3Gal1 and variants.

	Donor CMP-Neu5Ac			Acceptor Gal-β-1,3-GalNAc-α-*O*-Bn		
	K_M_ (µm)	k_cat_ (min^−1^)	k_cat_/K_M_ (mm^−1^ min^−1^)	K_M_ (µm)	k_cat_ (min^−1^)	k_cat_/K_M_ (mm^−1^ min^−1^)
ST3Gal1^a^	106 (±7)	201 (±5)	1896	26 (±1)	189 (±2)	7269
N147S (150)	668 (±159)^b^	75 (±9)	112	211 (±25)	48 (±2)	227
S148A (151)	481(±55)^b^	219 (±11)	455	105 (±12)	139 (±5)	1324
N170A (173)	184 (±29)	37 (±2)	201	96 (±16)	39 (±2)	406
Y191A (Y194)	876 (±206)^b^	38 (±5)	43	444 (±88)^b^	28 (±2)	63
Y230A (233)	205 (±18)	150 (±4)	732	854 (±105)^b^	178 (±11)	208
Y230F (233)	176 (±19)	162 (±6)	920	352 (±26)	208 (±5)	591
V315A (318)	180 (±23)	63 (±2)	350	1388 (±254)^b^	145 (±17)	105
V315Y (318)	271 (±51)	8 (±1)	28	549 (±78)^b^	12 (±1)	22
**C59S/C64S**						
(62–67)	121 (±13)	135 (±4)	1116	20 (±2)	137 (±3)	6850

Numbering corresponding to pST3Gal1 is given in parenthesis. Mean values and standard deviations of at least two replicates are shown.

^a^Values for wild-type MBP-ST3Gal1 were taken from^28^. ^b^For these variants saturation was not observed at the highest donor and acceptor concentrations employed (1.2 and 1.4 mm, respectively) (Supplementary Information, Figs. S5 and S6).

### Influence of mutations in the binding of donor and acceptor substrates

The homology model of hST3Gal1 using pST3Gal1 substrate bound structure (2wnb) as template proved a close overlapping of residues creating the binding sites of both enzymes (Supplementary Information, Fig. S1). Therefore, this model was employed to investigate the distances of selected residues to the donor and acceptor substrates and the possible effect of mutations on hST3Gal1 activity.

**Figure 2:**
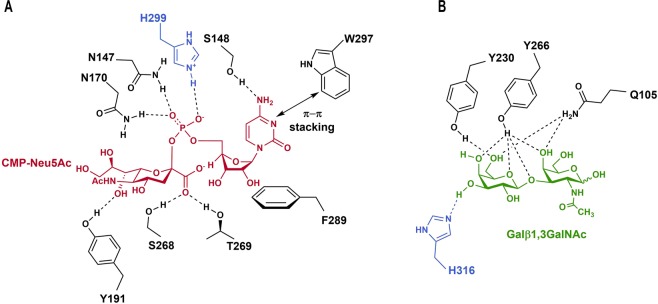
Residues involved in the recognition of donor CMP-Neu5Ac (**A**) and acceptor Galβ1,3GalNAc (**B**) by ST3Gal1 enzymes^12,13^. The catalytic acid H299 and catalytic base H316 are highlighted in blue.

Because GT29 enzymes share CMP-Neu5Ac as a common donor, the side chains involved in its binding are highly conserved. As previously observed for pST3Gal1^12,13^ and based on the hST3Gal1 model, residue W297 forms an aromatic stacking interaction with the cytidine moiety while S148 stabilizes the position of CMP by hydrogen bonding. The hydrophobic side of ribose interacts with F289 and further binding is achieved by hydrogen bonds between N147 and N170 with CMP phosphate group (Fig. 2). The side chain conformation of N173 (N170 in hST3Gal1) observed in the CMP bound crystal structures of pST3Gal1 and in hST3Gal1 model is transposed in the structures of other sialyltransferases, for example ST6Gal1 (4js2), ST8SiaIII (5bo6) and the sialyltransferase CstII from *Campylobacter jejuni* (1ro7). In those structures asparagine can establish hydrogen bonds with the donor via its side chain nitrogen. This conformation depicted in Fig. 2 is most likely adopted by N170 during catalysis in order to neutralize the negative charge of the leaving group CMP^12,17^. H299 acts as a hydrogen bond donor and most importantly as the catalytic acid, concomitantly activating phosphate by enhancing its quality as a leaving group^12,13^. Neu5Ac is engaged in polar interactions with Y191, S268 and T269 (Fig. 2).

In contrast to the conserved binding motif of the Neu5Ac donor, the substrate recognition site varies among sialyltransferases. This is expected due to the differing specificity for substrates and the formation of distinct glycosidic bonds. The substrate Gal-β-1,3-GalNAc-R is recognized by residues Q105 and Y266, which coordinate the C4-OH group of the GalNAc moiety. Additional hydrogen bonds are formed between the galactose C6-OH group and residues Y230 and Q105^12^. Residue Y266 furthermore contributes to the organization of the GalNAc moiety by polar interactions with the C4-OH group and the acetal function (Fig. 2). H316 contributes crucially to binding as well as catalysis of sialic acid transfer as it acts as a hydrogen bond acceptor and as the catalytic base, thus activating the galactose C3-hydroxyl group for the nucleophilic attack and stabilizing the transition state. In this S_N_2 like mechanism H299 and H316 act as acid and base catalysts, respectively (Fig. 2 and Supplementary Information, Fig. S7)^12,13^. Both side chains stabilize the transition state, subsequently leading to a one step inverting reaction and forming an α2,3 bond after release of the CMP leaving group.

**Figure 3:**
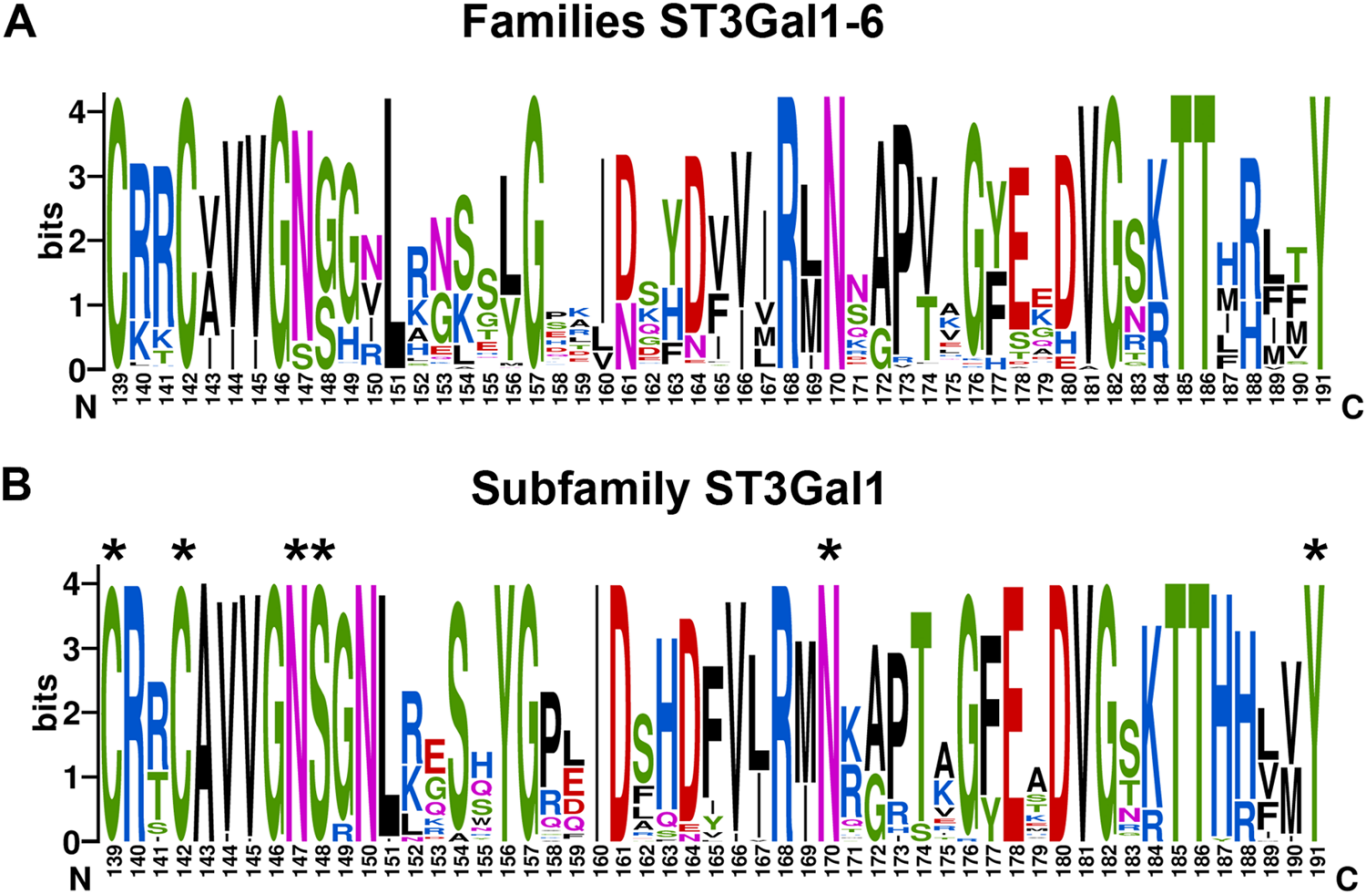
Conservation of sialylmotif L residues in the subfamily ST3Gal1-6 (**A**) and the subgroup ST3Gal1 (**B**). hST3Gal1 numbering is used. An asterisk indicates the residues that were mutated in this work. The graphical representation of sequence conservation was generated with WebLogo^47^ using 271 and 43 non redundant sequences from ST3Gal1-6 and ST3Gal1 enzymes, respectively.

Residues N147 and S148 interact exclusively with the donor substrate and are invariant in all characterized and predicted ST3Gal1 enzymes (Figs. 2 and 3). A strong hydrogen bond (2.7 Å) occurs between the δNH_2_ group of N147 and the phosphate group of CMP (Fig. 2 and Supplementary Fig. S8). CMP binds in the same conformation in the chemically different environment of all sialyltransferases with available crystal structure^17^; however, in most sequences from ST3Gal5 and ST6Gal1 a serine is found replacing asparagine at the position equivalent to N147. Although with a shorter side chain, serine is in principle able to coordinate the binding of CMP via its hydroxyl group. Substitution of asparagine by serine in variant N147S affected k_cat_ for donor and acceptor and increased the K_M_ for both substrates, showing no saturation at the assayed donor concentrations (Table 1 and Supplementary Fig. S5) and indicating that serine is unable to stabilize the donor binding in this enzyme.

**Figure 4:**
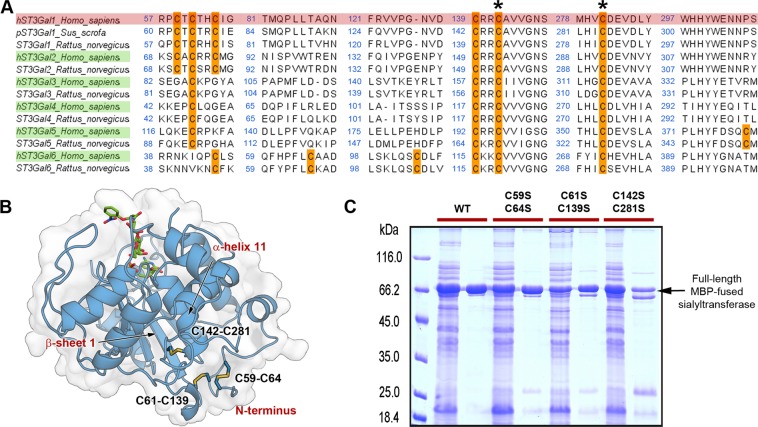
Position of disulphide bonds in subfamily hST3Gal1-6 and expression of hST3Gal1 disulphide bond variants. (**A**) Conservation of cysteine residues in subfamily ST3Gal1-6. Two asterisks indicate the conserved cysteines through the superfamily GT29. UniProt ID for sequences of human, porcine and rat sialyltransferases are shown in the next order: Q11201, Q02745, Q6H8N0, Q16842, Q11205, Q11203, Q2734, Q11206, P61131, Q9UNP4, Q68G12, Q9Y274 and P61943. (**B**) Location of disulphide bridges in hST3Gal1 (model). CMP and acceptor Gal-β-1,3-GalNAc-α-PhNO_2_ from PDB 2wnb are displayed to show the location of the active site. (**C**) Reducing SDS-PAGE (12% acrylamide) showing hST3Gal1 wild-type and disulphide bond variants in soluble cleared extracts and after purification by immobilized metal affinity chromatography. The band observed below the full length sialyltransferase corresponds to a slightly degraded hST3Gal1 with the C-terminal His-tag missing (Supplementary Information, Figs. S11 and 12).

In the subfamily ST3Gal1-6 only serine and glycine can be found at the position corresponding to S148 in hST3Gal1. While ST3Gal1 and ST3Gal2 enzymes have a serine, sialyltransferases from subgroups ST3Gal3-6 possess a glycine instead (Figs. 3 and 4). The exchange of serine by alanine eliminates the possibility to form a hydrogen bond between this residue and the amino group of the cytidine moiety. This change has a small effect on the k_cat_ for donor and acceptor, but affects K_M_ for CMP-Neu5Ac and Gal-β-1,3-GalNAc-α-*O*-Bn by 4.5- and 4-fold, respectively. Once again, saturation for the donor could not be observed at the measured CMP-Neu5Ac concentrations.

Residue N170 interacts with the phosphate group of CMP and can form a hydrogen bond with the C8-OH group of Neu5Ac, as observed from the structural alignment of porcine (2wnb) and human (model) ST3Gal1 with the sialyltransferase CstII from *Campylobacter jejuni* bound to CMP3F-Neu5Ac (PDB 1ro7) (Fig. 1 and Supplementary Fig. S8). N170 is entirely conserved through family ST3Gal1-6 (Fig. 3). Expression and purification of variant N170A were rather inefficient and unsurprisingly, substitution of N170 by alanine had a great impact on k_cat_ for donor and acceptor molecules, as well as on K_M_.

The contribution of residues Y191 and Y230 to catalysis was previously analysed in corresponding variants Y194F and Y233A from pST3Gal1^12^. Y191 is fully conserved in subfamily ST3Gal1-6 and its phenolic hydroxyl group forms a hydrogen bond with the C7-OH group of Neu5Ac and stacking interactions with the galactosyl-moiety of the acceptor (Figs. 1 and 2). Substitution of Y194 by phenylalanine in pST3Gal1 decreases the variant’s activity on the donor by around 15-fold as a consequence of the removal of said hydrogen bond, leading to a reduction in the binding transition state stabilization of 1.7 kcal mol^−1^ ^12^. A more drastic effect was observed in this work for the modification Y191A in hST3Gal1 (Table 1), in particular for the acceptor. The exchange by alanine prompted naturally a severe decrease in k_cat_, since the stacking interaction with galactose is removed. The reduction in the transition state stabilization corresponds to 2.3 and 2.9 kcal mol^−1^ for donor and acceptor, respectively (Supplementary Information, Table S1). Substrate saturation was not achieved for the variant, neither for the donor nor for the acceptor.

Residue Y230 stacks with the *N*-acetylgalactosamine moiety of the acceptor and forms a hydrogen bond with the galactose C6-OH group (Figs. 1 and 2). Stacking is preserved when Y230 is exchanged by phenylalanine and thus only moderate changes are observed in the kinetic parameters of variant Y230F for CMP-Neu5Ac when compared to the wild-type activity (Table 1). Conversely, the loss of the hydrogen bond between galactose and the hydroxyl group of tyrosine reduces the catalytic efficiency for the acceptor by approximately 12-fold. Removal of both, hydrogen bonding and stacking interactions in variant Y230A causes a 35-fold activity reduction on the acceptor, a result comparable to a similar mutation (Y233A) in pST3Gal1, where a decrease in transition state stabilization of 1.2 and 2.1 kcal mol^−1^ for donor and acceptor was observed^12^. These results are in agreement with the values obtained for hST3Gal1 variant Y230A, as the removal of hydrogen bonding and stacking interactions causes a decrease in the transition state stabilization of 0.6 kcal mol^−1^ for the donor and 2.2 kcal mol^−1^ for the acceptor (Supplementary Information, Table S1).

β-galactoside α-2,6-sialyltransferase 1 (ST6Gal1) transfers Neu5Ac to the C6-hydroxyl group of a disaccharide motif containing a terminal galactose. Structurally ST3Gal1 and ST6Gal1 share a central core of about 200 amino acids, but differ in the surrounding loops, including an extra α-helix in ST6Gal1 that inverts its C-terminal β-strand with regard to ST3Gal1^13,17^ (Supplementary Information, Fig. S3). A clear difference between these two enzymes is the network of interactions that provide an optimal binding and orientation for the acceptor substrates, thus contributing to their regioselectivity. Residue Y369 is part of said network of contacts in enzyme ST6Gal1. Residue V315 from hST3Gal1 occupies a similar position to Y369 (Supplementary Information, Fig. S3). The mutation V315A affects drastically the functionality of hST3Gal1, decreasing the activity for donor and acceptor by 18-and 70-fold, respectively, when compared to the wild-type enzyme. The mutation most likely impacts the fine tuning of the catalytic base H316 regarding to the substrates, demonstrating that small changes in H316 vicinity have a significant effect on activity. As expected, the exchange of valine by tyrosine had a greater impact on activity. The mutation possibly introduces a steric hindrance between V315Y and Y266 (a residue interacting with the GalNAc unit), or directly interferes with the optimal binding of Gal-β-1,3-GalNAc-α-*O*-Bn by reducing the size of the binding cavity. Both possibilities are in agreement with the low k_cat_/K_M_ values obtained for this variant (Table 1) and are supported by the lack of substrate saturation resulting in a high K_M_ for the acceptor.

The regioselectivity of variant V315Y (the formation of an α-2,3 linkage in the product) remained unaltered (Supplementary Information, Fig. S9). As the wild-type enzyme, V315Y was unable to use *N*-acetyllactosamine (LacNAc) efficiently as acceptor to produce either 3- or 6-sialyl-LacNAc (Supplementary Information, Fig. S9). Although the LacNAc motif is the best acceptor for ST6Gal enzymes, the absence of the axial C4-OH group in substrates containing a GlcNAc- instead of the GalNAc-moiety is responsible for the disruption of important contacts between ST3Gal1 enzymes and these kind of acceptors, resulting in poor activities (500-fold lower k_cat_/K_M_ values)^12^.

Up to now, a switch in regioselectivity from α-2,3 to α-2,6 has only been achieved for bacterial sialyltransferases, which belong to GT80. Such change occurred by introducing a couple of amino acid substitutions that prompted the binding of the acceptor in a favourable conformation to receive the sialic acid at the galactose C6-OH group in lactose and LacNAc acceptors^32,33^. In human sialyltransferases substrate binding and regioselectivity seem to be controlled by structural elements that greatly differ in ST6Gal1 and ST3Gal1 enzymes, such as a large N-terminal extension in the catalytic domain of the former that is absent in the latter proteins^17^ (Supplementary Information, Fig. S3). Thus, a switch in the regioselectivity of GT29 sialyltransferases is predicted to necessitate a major acceptor binding site remodelling.

### Contribution of disulphide bonds to activity and stability

The role of family- and subfamily-conserved disulphide bonds has been thoroughly analysed for ST6Gal1^14,34,35^. *In vivo* experiments demonstrated that mutations of cysteines 181 and 332 (corresponding to C142 and C281 in hST3Gal1) abrogates the activity of hST6Gal1 and induces protein misfolding, impeding therefore the transport of the enzyme from the endoplasmic reticulum to the Golgi apparatus^34^. While essential for activity, mutation of the disulphide formed between C353 and C364 does not affect the protein localization. Interruption of the bond C142-C406 preserves both activity and localization^35^. Studies regarding the implication of ST3Gal1 disulphide bonds in activity, stability and cellular localization *in vivo* are missing.

We analysed the *in vitro* effect of suppressing each one of the three disulphide bonds of hST3Gal1 by exchanging cysteine by serine residues in double variants C59S-C64S, C61S-C139S and C142S-C281S. hST3Gal1 and hST3Gal2 enzymes share an amino acid identity of 49.3% and the same pattern and spatial location of disulphide bonds, based on multiple alignments and modelling of hST3Gal2 with pST3Gal1 as template (not shown). ST3Gal1 and ST3Gal2 have as well overlapping acceptor specificity on O-linked glycans, although ST3Gal2 is primarily responsible for terminal sialylation of gangliosides GD1a and GT1b^11,36^. ST3Gal3 and ST3Gal4 enzymes also display the same disulphide arrangement, while ST3Gal5 and ST3Gal6 enzymes show a unique disulphide distribution when compared to other members of the ST3Gal subfamily (Fig. 4A). In agreement with previous studies, the removal of cysteine residues C142 and C281 (variant C142S-C281S) abolishes the enzyme’s activity (Supplementary Information, Fig. S10). C142 is located in the β-sheet 1 in the sialylmotif L and C281 is found in a loop that precedes the α-helix 11 in the siaylmotif S^13^ (Fig. 4B). These structural elements bring together the scaffold that generates the CMP-Neu5Ac binding site. According to the structural model, disulphide C61-C139 is found in a disordered loop at the hST3Gal1 N-terminal region, spatially close to C59S-C64S. Activity of variant C61-C139 could not be detected neither using the cleared lysate nor purified protein (Supplementary Information, Fig. S10). Cysteine 139 is located in a loop that antecedes the β-sheet 1, thus disulphide C61-C139 probably contributes to the stabilization of this β-sheet in relation to α-helix 11.

Finally, removal of disulphide C59S-C64S has a lesser impact on activity, as the variant retains around 70% of the wild type activity (k_cat_) for donor and acceptor (Table 1).

We investigated whether the removal of a cysteine pair would affect the formation of the remaining disulphides by submitting the MBP-fusion proteins hST3Gal1 and variants C59S-C64S and C61-C139 to NanoLC-MS/MS analysis (Supplementary Information, Figs. S13–15). Cysteine residues not involved in disulphide bonds were labelled with *N*-ethylmaleimide before sample reduction with TCEP (tris (2-carboxyethyl) phosphine). After reduction, cysteines were alkylated with NEM-D5. The wild-type sialyltransferase and both variants show similar profiles regarding to the presence/absence of free cysteines in differential cysteine labelling experiments, indicating that probably the exclusion of one disulphide bridge does not influence the occurrence of the other two native cysteine pairs.

**Figure 5:**
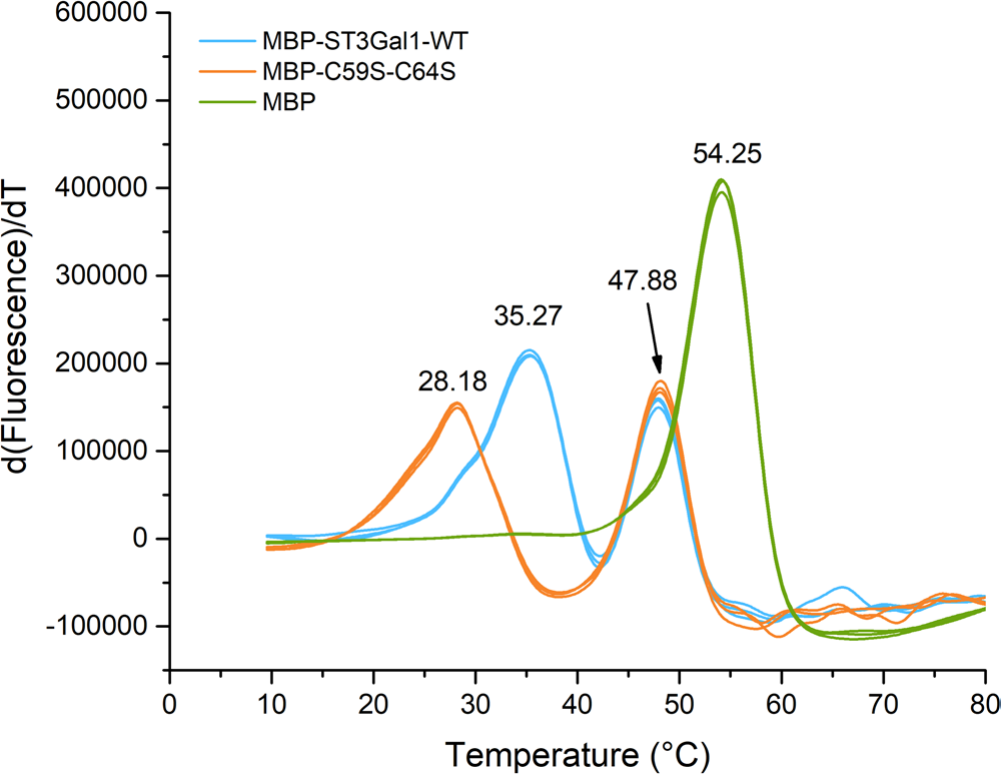
Melting temperature (T_m_) of maltose binding protein and MBP-fused hST3Gal1-WT and variant C59S-C64S. Tm values (°C) corresponding to each protein are displayed.

Finally, the contribution of the intra molecular bond C59-C64 to the protein thermal stability was also examined. The melting temperature (T_m_) of the MBP-fused wild-type enzyme and variant C59S-C64S was analysed via differential scanning fluorimetry^37^. Two thermal denaturation curves were clearly identified for solubility enhancer MBP and the sialyltransferases. The absence of disulphide C59-C64 reduces the T_m_ of variant C59S-C64S by 7 °C (Fig. 5).

Non fused MBP displays a higher T_m_ than that observed in fusions with the sialyltransferases because it is stabilized by substrate binding. While MBP-fused sialyltransferases were purified via a hexa-histidine-tag, non-fused MBP was purified over an amylose resin and eluted with maltose, which remains bound to the protein. Binding of maltose to MBP was reported to raise the T_m_ in the range of 8–15 °C, depending on the buffer conditions^38^. In this work we observed an increase of 6.3 °C (Fig. 5). To the best of our knowledge, data regarding the thermal stability of heterologously expressed non-glycosylated mammalian sialyltransferases are not available.

## Conclusions

Sialic acids play multiple biological roles in mammals. For instance, they are involved in immunology, hereditary diseases and cancer progression^21,39^. The beta-galactoside alpha-2,3-sialyltransferase 1 (ST3Gal1) is upregulated in cancer and thus it is central to a strategy focused on the synthesis of inhibitors against hypersialylation of proteins and lipids to prevent metastasis and overcome resistance to chemotherapy^21^. Structural and biochemical studies on this and other sialyltransferases are needed in order to assist the rational design of potent and selective inhibitors.

Here we exchanged various residues of hST3Gal1 that form the binding sites for donor CMP-Neu5Ac and the acceptor motif Gal-β-1,3-GalNAc-R to analyse their influence on catalysis. Residues were selected employing the crystallographic structure of pST3Gal1 as a template for structural modelling of its human homolog hST3Gal1^13^. Binding sites for both substrates are constituted by the catalytic base H316 and amino acids Q105, Y191, Y230, Y266, T269, W297, H299, F310, R311, K312, whose contribution to catalysis was earlier reported for pST3Gal1^12^. We explored the effect of mutating residues Y191 and Y230 in hST3Gal1, confirming the importance of the hydrogen bonds provided by their phenolic hydroxyl groups as well as the stacking interactions for an optimal donor and/or substrate binding. The outcome of mutating amino acids N147, S148 and N170, which interact exclusively with the donor was also analysed, observing that activities toward both donor and acceptor are drastically decreased for variants N147S and N170A.

The involvement of subfamily-specific disulphide bonds in the proper folding and activity of sialyltransferases ST6Gal1, ST8Sia1, ST8Sia4 and ST8Sia6 is well documented^14,34,40–42^; however, information regarding ST3Gal1 enzymes is scarce. Here it was demonstrated that disulphides C61-C139 and C142-C281 (which is entirely conserved in GT29 enzymes) are essential for activity of hST3Gal1. Although disulphide C59-C64 is not strictly needed for activity, its removal has a detrimental impact on the protein thermal stability.

## Materials and Methods

### Materials

Nucleoside Monophosphate Kinase (NMPK) was obtained from Roche (Germany). CMP-Neu5Ac disodium salt and benzyl 2-acetamido-2-deoxy-3-*O*-(β-d-galactopyranosyl)-α-d-galactopyranoside (Gal-β-1,3-GalNAc-α-*O*-Bn) were acquired from Carbosynth (UK). Lactate dehydrogenase/pyruvate kinase solution (PK/LDH) and asialofetuin were obtained from SIGMA (Germany). NADH and phosphoenolpyruvic acid monopotassium salt (PEP) were purchased from Alfa AppliChem (Germany) and Aesar (Germany), respectively. Electrophoresis and other reagents were from Merck and SIGMA.

### Cloning, expression and purification of hST3Gal1 and variants

Synthetic gene encoding hST3Gal1 (Uniprot code Q11201) lacking the transmembrane region (starting in E45) and with optimized codon usage for expression in *E. coli* was obtained from GeneArt (Life technologies, Germany). The gene includes the restriction sites *NdeI* and *BamHI* at the 3′ and 5′ ends respectively and was cloned into the vector pMAL-c5X (New England BioLabs, USA). This plasmid was used as a template to generate single and double mutants employing the Quikchange lightning and Quikchange lightning Multi site-directed mutagenesis kits (Stratagene, USA), respectively. Variant C59S-C64S was generated by inverse mutagenesis. Primers used to generate the variants are shown in Table 2 (SIGMA, Germany).

**Table 2:** Primers for site directed mutagenesis.

Position in pST3Gal1	Mutation in hST3Gal1^a^	Primers sequence (5′to 3′)
N150	N147S	Forward: GCTGTAGTAGGCTCTAGCGGGAATCTG
		Reverse: CAGATTCCCGCTAGAGCCTACTACAGC
S151	S148A	Forward: GTAGTAGGCAATGCAGGGAATCTGCGTG
		Reverse: CACGCAGATTCCCTGCATTGCCTACTAC
N173	N170A	Forward: GTCCTGCGTATGGCAAAAGCCCCGACTG
		Reverse: CAGTCGGGGCTTTTGCCATACGCAGGAC
Y194	Y191A	Forward: CCATCATCTGGTTGCGCCTGAGAGCTTTC
		Reverse: GAAAGCTCTCAGGCGCAACCAGATGATGG
Y233	Y230A	Forward: CATCTCACACACTGCGATCCCTGTGCCGG
		Reverse: CCGGCACAGGGATCGCAGTGTGTGAGATG
	Y230F	Forward: CATCTCACACACTTTTATCCCTGTGCCGG
		Reverse: CCGGCACAGGGATAAAAGTGTGTGAGATG
V318	V315A	Forward: CGTAAAACCGGCGCGCACGATGCTG
		Reverse: CAGCATCGTGCGCGCCGGTTTTACG
	V315Y	Forward: CTTTTCGTAAAACCGGCTATCACGATGCTGATTTC
		Reverse: GAAATCAGCATCGTGATAGCCGGTTTTACGAAAAG
C62-C67	C59S-C64S^b^	Forward: CCTTCTACGTGTACTCACTCTATTGGTCAGCGTAAACTG
		Reverse: ACGGTGTTTAATCAGACGTTTCAGGTTCTCGCTCAG
C64-C142	C61S-C139S	Forward C61S: CCGTCCTTGTACGTCTACTCACTGTATTGGTC Forward C139S: CGTTCTGTGGGTTCCCGCCGTTGTGC
C145-C284	C142S-C281S	Forward C142S: GGTTGCCGCCGTTCTGCTGTAGTAGGC
		Forward C281S: CTCCATGCATGTCTCTGACGAGGTGGATCTG

^a^Numbers in model are based on the full-length sequence. ^b^Whole plasmid amplification was performed by inverse PCR with phosphorylated primers; the forward primer contains the double mutation.

*E. coli* Origami2 (DE3) (Novagen, Germany) carrying plasmid pMJS10 (kindly provided by Prof. Ruddock, University Oulu, Finland) was transformed with pMAL-c5X carrying the wild type hST3Gal1 or its variants. pMJS10 contains the genes that encode for the enzymes sulfhydryl oxidase and disulphide isomerase (Erv1p and DsbC)^29^. Sialyltransferases were expressed as N-terminus MBP- and C-terminus His-tagged-proteins as previously reported^28^. Briefly, 5 mL overnight cultures started from one colony of freshly transformed *E. coli* were used to inoculate 0.5 L LB broth with 12.5 µg mL^−1^ of tetracycline, 200 µg mL^−1^ of ampicillin and 30 µg mL^−1^ of chloramphenicol added. Expression of Erv1p/DsbC was induced at 30 °C at an OD_600_ of 0.3–0.4 by adding l-(+)-arabinose to a final concentration of 0.3%. Temperature was decreased to 17 °C when the cultures reached an OD_600_ of 0.5–0.6 and the expression of sialyltransferases was induced during 22 h with IPTG to a final concentration of 0.1 mm. Cells were harvested by centrifugation and the pellet was resuspended in 8 mL lysis buffer (25 mm NaH_2_PO_4_, 25 mm Na_2_HPO_4_, 0.3 m NaCl and 20 mm imidazole, pH 8) and frozen at −20 °C. Thawed cells were disrupted by sonication. The lysate was centrifuged at 4 °C and 16,000 g for 30 minutes and the cleared supernatant was filtered, diluted to 4 mg mL^−1^ total protein and loaded onto a gravity column containing His-Select nickel affinity gel (SIGMA, Germany) previously equilibrated with lysis buffer (25 mm NaH_2_PO_4_, 25 mm Na_2_HPO_4_, 0.3 m NaCl and 20 mm imidazole, pH 8). After 1 h incubation at 4 °C, the column was washed with 20 mL of lysis buffer added with 10% glycerol. The protein was eluted in a single step with 2.5 mL elution buffer (25 mm NaH_2_PO_4_, 25 mm Na_2_HPO_4_, 0.3 m NaCl and 250 mm imidazole, pH 8). Purified proteins were buffer exchanged against 50 mm HEPES, pH 7.5 or 50 mm Soerensen’s buffer and concentrated on Vivaspin-500 ultrafiltration tubes with a MWCO of 50,000 Da (Sartorius, Germany). Expression and purification of sialyltransferases were analysed by SDS-PAGE loading crude lysates, cleared supernatants and purified proteins onto 12% acrylamide gels.

### Protein modelling

Two homology models of hST3Gal1 were generated with the SWISS-MODEL server^22^ using the pST3Gal1 apo- (PDB 2wml) and substrate-bound (2wnb) structures as templates. Alignment of templates and hST3Gal1 models was performed with Chimera (http://www.rbvi.ucsf.edu/chimera), reporting a Q-score of around 0.96 for both models. Identical structures would have a Q-score of 1.0. RMSD values for mutagenized residues N147, S148, N170, Y191, Y230 and V315 are shown in Supplementary Information, Fig. S1.

### Activity assay

A continuous coupled spectrophotometric assay was employed to determine hST3Gal1 initial rates on donor and acceptor substrates. One unit is defined as the amount of protein that transfers 1 µmol of Neu5Ac from CMP-Neu5Ac to Gal-β-1,3-GalNAc-α-*O*-Bn or asialofetuin per min at 37 °C, in 50 mm HEPES, pH 7.4. The multi-enzymatic assay is based on NADH oxidation via the coupled reactions of sialyltransferase, NMPK, PK and LDH^43^. The concentration of the acceptor Gal-β-1,3-GalNAc-α-*O*-Bn was varied from 0.005 to 1.4 mm, while keeping the donor (CMP-Neu5Ac) concentration constant to 0.7 mm. Additionally, the concentration of the donor was modified from 0.01 to 1.2 mm while maintaining the concentration of Gal-β-1,3-GalNAc-α-*O*-Bn to 1 mm. Kinetic parameters were obtained at 37 °C using a GENios plate reader (Tecan, Switzerland). 100 µL reactions were performed on a 384-well microtiter plates containing 50 mm HEPES, pH 7.4, 0.7 mm PEP, 0.27 or 0.29 mm NADH, 2 mm ATP, 50 mm KCl, 10 mm MnCl_2_, 10 mm MgCl_2_, 1 g L^−1^ BSA, 15 mU of NMPK, 8 U of PK, 12 U of LDH and variable concentrations of donor and acceptor. Microtiter plates were centrifuged at 1000 g during 30 s and reactions were incubated at 37 °C for 22 min to deplete the CMP present in the donor solution and NDPs from the ATP and NMPK solutions^12^. Incubation of the reaction components before adding hST3Gal1 is also required to obtain the rate of CMP-Neu5Ac spontaneous hydrolysis. After this period of incubation, 2 µL of sialyltransferase solution was added, the microtiter plate was centrifuged at 1000 g for 30 s and the change in absorbance (340 nm) was measured during 42 minutes at 37 °C. An extinction coefficient for NADH of 6220 m^−1^ cm^−1^ and a path length of 0.82 cm were considered for initial rates calculations. At least two replicates were performed for each substrate/acceptor concentration.

K_M_ and k_cat_ values were obtained in OriginPro (OriginLab) by using nonlinear least squares and the Michaelis-Menten equation from fitting a set of rate versus substrate concentration values. Mean values and standard deviations (±SD) were calculated from these measurements.

The impact of mutations on the global catalytic efficiency in terms of the transition state destabilization was calculated using the following equation that incorporates kinetics and thermodynamics parameters^12,44^:

$$\Delta \Delta {G}^{\#}=2.303\,{\rm{RT}}\,\log \,{({{\rm{k}}}_{{\rm{cat}}}/{{\rm{K}}}_{{\rm{M}}})}_{{\rm{WT}}}/{({{\rm{k}}}_{{\rm{cat}}}/{{\rm{K}}}_{{\rm{M}}})}_{{\rm{mutant}}}$$

### Synthesis of sialyl-products and analysis by HPAEC-PAD

Samples containing cleared cell lysates with expressed sialyltransferases (50 ng total protein µL^−1^) or purified enzymes (1 ng µL^−1^), Gal-β-1,3-GalNAc-α-*O*-Bn (0.5 or 2.0 mm) or LacNAc (2.0 mm) and CMP-Neu5Ac were prepared in 20.0 mm MOPS, pH 7.5 in 85.0 μL final volumes. For variant C142S-C281S, the double amount of total (cleared extract) or purified protein was used. Reactions were incubated at 37 °C and samples were taken after 1 or 4 h reaction time. Substrate consumption and synthesis of sialosides were analysed by High-Performance Anion Exchange Chromatography (HPAEC) with a Dionex ICS-5000 + SP system utilizing a Carbopac PA10 column (250 × 2 mm) at a flow of 250 μL min^−1^. 100 mm NaOH (A), 100 mm NaOH containing 1 m NaOAc (B) and 250 mm NaOH (C) were used as eluents. Acceptor molecules and sialosides were resolved using a multistep gradient programmed as follows: 0–5 min 100% A, 5–20 min 0–20% B, 20–21 min 20–45% B, 21–28 min 45% B, 28–35 min 100% C, 35–45 min 100% A. Acceptors LacNAc and Gal-β-1,3-GalNAc-α-*O*-Bn and sialosides 6-sialyl-LacNAc and 3′-sialyl-Gal-β-1,3-GalNAc-α-*O*-Bn were identified using appropriate standards.

### Circular dichroism spectroscopy

CD spectra of the fusion hST3Gal1wild type and variants were recorded with a JASCO model J-810 spectropolarimeter. The measurements were performed at 20 °C employing a protein concentration of 0.8–1.2 µm in 50 mm Soerensen’s buffer, pH 6.5. CD spectra were recorded over the range of 190 to 280 nm with a response of 0.5 s, a data pitch of 0.1 nm and a scanning speed of 100 nm min^−1^. Signal to noise ratio was reduced by averaging 10 accumulations. The spectrum of Soerensen’s buffer was subtracted.

### Differential scanning fluorimetry (DSF)

100 μL samples containing a final concentration of 2 μm protein (ST3Gal1-wild-type and variant C59S-C64S fused with the maltose binding protein (MBP), as well as MBP alone), 50 mm HEPES buffer pH 7.4, 100 mm NaCl and 10 × Sypro Orange (Thermo Fisher, Germany) were prepared in 0.5 mL Eppendorf tubes. Control experiments without protein were also included. Aliquots of 20 μL of each sample were applied 3 times on a 96 well plate that was later sealed with a transparent adhesive film and centrifuged at 1000 g for 1 minute. Samples were maintained in ice during preparation. The well plate was placed in a QuantStudio™ 5 Real-Time PCR System (ThermoFisher Scientific, Germany) and changes in fluorescence over a temperature range of 10–95 °C were recorded using the ROX filter and a temperature ramp of 0.02 °C s^−1^.

Fluorescence data were processed with the Protein Thermal Shift™ Software to obtain melting temperature values.

The experiments were performed with two different batches of purified proteins to demonstrate the presence of homogenous protein preparations.

### Differential cysteine labelling

*N*-ethylmaleimide (NEM) labelling was performed according to the protocol described by McDonagh *et al*.,^45^ with minor modifications. Briefly, 15 µL 200 mm NEM (Sigma) in 100 mm HEPES, pH 7.4, was added to 5 µg 15 µL^−1^ protein in HEPES and stored overnight at 4 °C. SDS was added to a final concentration of 1%. After 2 h at 37 °C protein precipitation was performed according to Wessel and Fluegge with chloroform/methanol^46^. The precipitated protein was washed 2 times with 400 µL methanol:chloroform (3:1, v/v). The protein pellet was dissolved in 10 µL 1% SDS in HEPES and heated to 95 °C for 5 min. Reduction was performed with 1.1 µL 500 mm Bond-Breaker TCEP (Thermo Scientific) for 5 min at 95 °C. Reduced cysteine residues at this state were alkylated with 10 µL 200 mm NEM-D5 (Euriso-Top) in HEPES for 2 h at room temperature.

### Gel electrophoresis and in-gel digestion

NuPAGE LDS sample buffer (Life Technologies) was added and separation was performed on NuPAGE Novex 4–12% Bis-Tris gels (Life Technologies) with MOPS buffer according to manufacturer’s instructions. Gels were washed three times for 5 min with water, stained for 45 min with Simply Blue™ Safe Stain (Life Technologies) and washed with water for 1 h.

Gel bands were excised and destained with 30% acetonitrile in 0.1 m NH_4_HCO_3_ (pH 8), shrunk with 100% acetonitrile, and dried in a vacuum concentrator (Concentrator 5301, Eppendorf, Germany). Digests were performed with 0.1 µg trypsin or elastase per gel band overnight at 37 °C in 0.1 m NH_4_HCO_3_ (pH 8). After removing the supernatant, peptides were extracted from the gel slices with 5% formic acid, and extracted peptides were pooled with the supernatant.

### NanoLC-MS/MS analysis and MS data analysis

NanoLC-MS/MS analyses were performed on a LTQ-Orbitrap Velos Pro (Thermo Scientific) equipped with a PicoView Ion Source (New Objective) and coupled to an EASY-nLC 1000 (Thermo Scientific). Peptides were loaded on capillary columns (PicoFrit, 30 cm × 150 µm ID, New Objective) self-packed with ReproSil-Pur 120 C18-AQ, 1.9 µm (Dr. Maisch) and separated with a 30-minute linear gradient from 3% to 30% acetonitrile and 0.1% formic acid and a flow rate of 500 nL/min. MS scans were acquired in the Orbitrap analyser with a resolution of 30,000 at m/z 400, MS/MS scans were acquired in the Orbitrap analyser with a resolution of 7,500 at m/z 400 using HCD fragmentation with 30% normalized collision energy. A TOP5 data-dependent MS/MS method was used; dynamic exclusion was applied with a repeat count of 1 and an exclusion duration of 30 seconds; singly charged precursors were excluded from selection. Minimum signal threshold for precursor selection was set to 50,000. Predictive AGC was used with AGC target a value of 1e6 for MS scans and 5e4 for MS/MS scans. Lock mass option was applied for internal calibration in all runs using background ions from protonated decamethylcyclopentasiloxane (m/z 371.10124).

Database search was performed against a small in-house database containing the protein sequences of interest with PEAKS 10 software (Bioinformatics solution Inc.) with the following parameters: peptide mass tolerance: 10 ppm, MS/MS mass tolerance: 0.015 Da, enzyme: “none”; variable modifications: Acetylation (Protein N-term), Pyro-glu from Q, *N*-ethylmaleimide on cysteines, D5 *N*-ethylmaleimide on cysteines. Results were filtered to 1% PSM-FDR by target-decoy approach.

## Supplementary information

Supplementary Information

## Data Availability

All data generated or analysed during this study are included in this published article (and its Supplementary Information files).
