# Supplementary material for: Structural and functional role of disulphide bonds and substrate binding residues of the human beta-galactoside alpha-2,3-sialyltransferase 1 (hST3Gal1)
Source: Sci Rep. 2019 Nov 29;9:17993. doi: 10.1038/s41598-019-54384-8 (PMC6884586; doi:10.1038/s41598-019-54384-8)
Supplement: Supplementary file 1 — Supplementary Information [file 41598_2019_54384_MOESM1_ESM.pdf]

Structural and functional role of disulphide bonds and substrate binding residues of the human beta-galactoside alpha-2,3-sialyltransferase 1 (hST3Gal1)

Institut für Organische Chemie, Julius-Maximilians-Universität, Am Hubland, 97074 Würzburg, Germany

seibel@chemie.uni-wuerzburg.de

Maria Elena Ortiz-Soto[1], Sabine Reising[1], Andreas Schlosser[2] and Jürgen Seibel[1*]

[1] Institut für Organische Chemie, Universität Würzburg, Am Hubland, 97074 Würzburg, Germany.

[2] Rudolf-Virchow-Zentrum für Experimentelle Biomedizin, Universität Würzburg, Josef-Schneider Str. 2, Haus D15, 97080 Würzburg, Germany

* Corresponding author

seibel@chemie.uni-wuerzburg

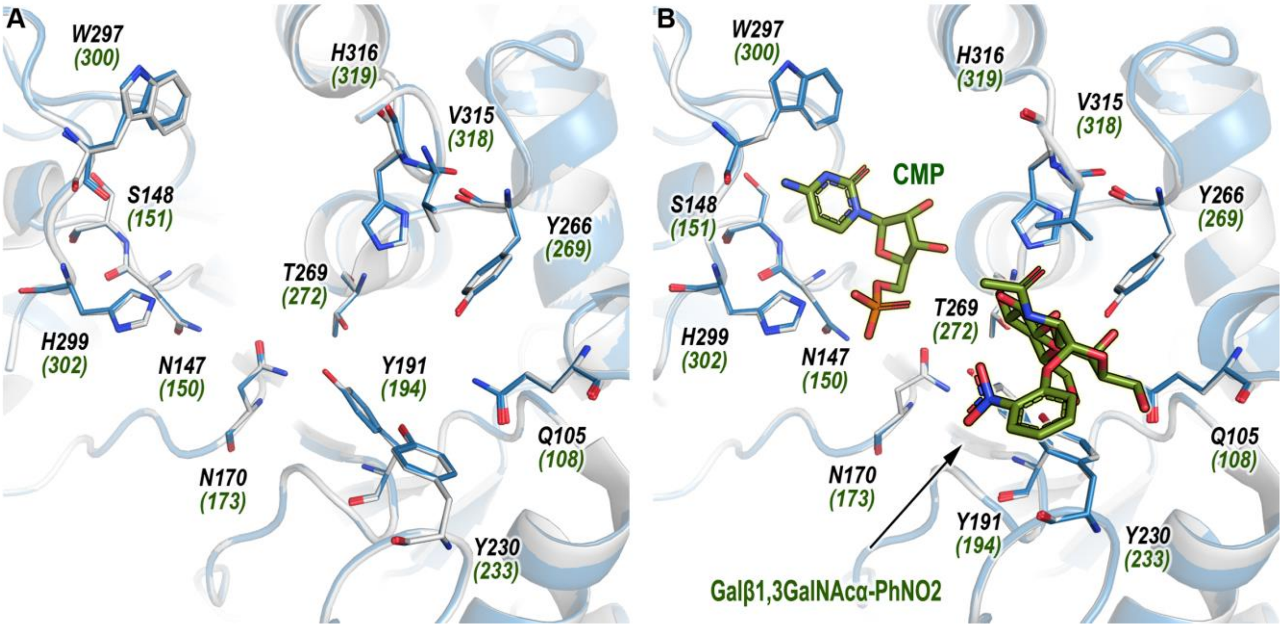

C

RMSD 2wml chain A (apo) and hST3Gal1 model (Evaluating superpositions across all 274 fully populated columns in the final alignment: Overall RMSD: 0.073; SDM (cutoff 5.0): 1.463; Q-score: 0.961)				
**Residue number**		**CA**	**Backbone**	**Full residue**
**Porcine**	**human**			
Q108	105	0.047212	0.099111	0.109525
N150	147	0.065322	0.071151	0.079397
S151	148	0.041243	0.115371	0.101617
N173	170	0.071477	0.094189	0.092601
Y194	191	0.048580	0.066791	0.053670
Y233	230	0.031177	0.044272	0.058212
Y269	266	0.077531	0.200214	0.137348
T272	269	0.045978	0.107869	0.084468
W300	297	0.492554	1.495610	0.868496
H302	299	0.073437	0.090341	0.084497
V318	315	0.047550	0.076243	0.063040
H319	316	0.184187	0.325028	0.210042
RMSD 2wnb chain A (substrate-bound) and hST3Gal1 model (Evaluating superpositions across all 272 fully populated columns in the final alignment: Overall RMSD: 0.063; SDM (cutoff 5.0): 1.270; Q-score: 0.957)				
Q108	105	0.058447	0.075135	0.096793
N150	147	0.079718	0.090803	0.086015
S151	148	0.035903	0.122748	0.115048
N173	170	0.088826	0.148593	0.120225
Y194	191	0.070150	0.106469	0.088033
Y233	230	0.082934	0.105799	0.079829
Y269	266	0.029682	0.145133	0.102836
T272	269	0.026571	0.076143	0.065258
W300	297	0.044553	0.078342	0.060341
H302	299	0.058112	0.082806	0.097150
V318	315	0.046184	0.086554	0.095002
H319	316	0.079272	0.135295	0.106292

Supplementary Figure S1. Structural alignment of human and porcine ST3Gal1. Model of hST3Gal1 (blue, SWISS-MODEL server) using A) the pST3Gal1 apo-structure (white, PDB 2wml) and B) the

pST3Gal1 substrate-bound structure (white, PDB 2wnb) as a template. The numbering corresponding to the human and porcine enzymes is in black and green font, respectively. C) RMSD values of the structural alignments performed with UCSF Chimera (http://www.rbvi.ucsf.edu/chimera). The tool Match -> Align with a cutoff of 5.0 Å and without iterate superposition/alignment cycles was employed. RMSD: CA - using one point (alpha carbon) per residue. RMSD: backbone - based on peptide backbone atoms (N, CA, C, O). RMSD: full - for residues of the same name, based on all distances between atoms of the same name (without correcting for symmetries between equivalent atoms with different names, e.g., OD1 and OD2 in aspartic acid). Structural Distance Measure (SDM) SDM is zero for identical structures and increases as the similarity decreases. The Q-score is zero for completely dissimilar or not superimposed structures and one for identical structures.

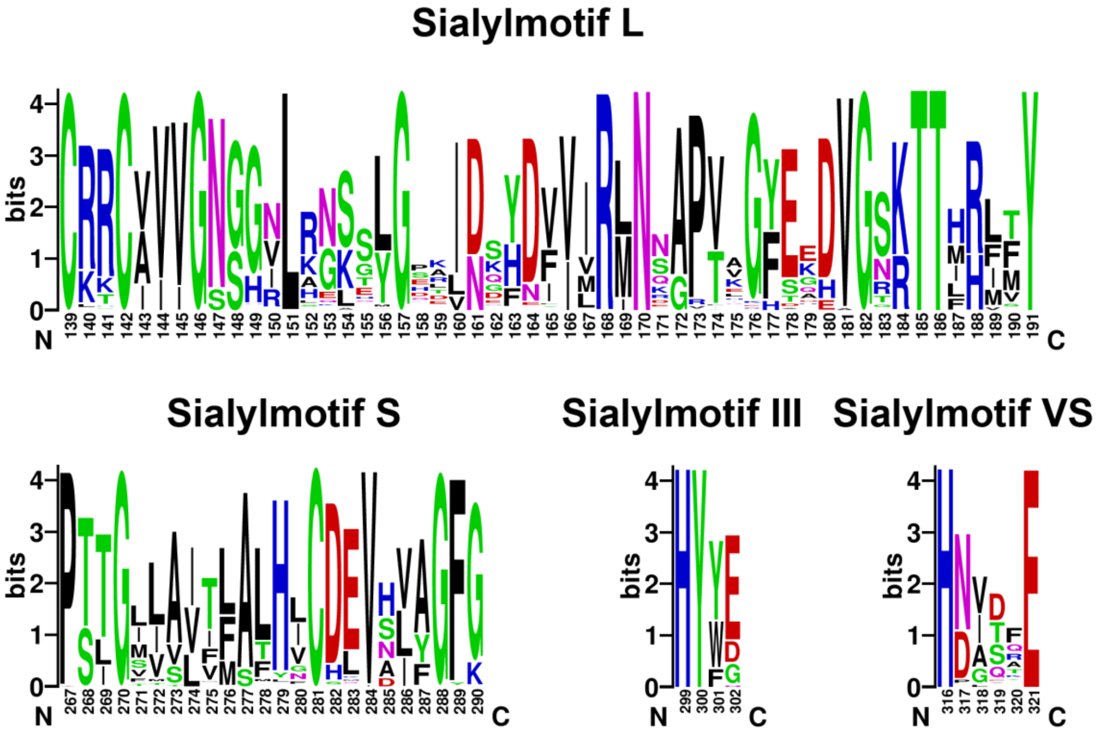

Supplementary Figure S2. Sequence conservation of sialylmotifs L, S, III and VS in families ST3Gal1-6. The graphical representation was generated with WebLOGO (University of California, Berkeley)[1]. 271 non-redundant sequences of ST3Gal1-6 enzymes were employed for analysis.

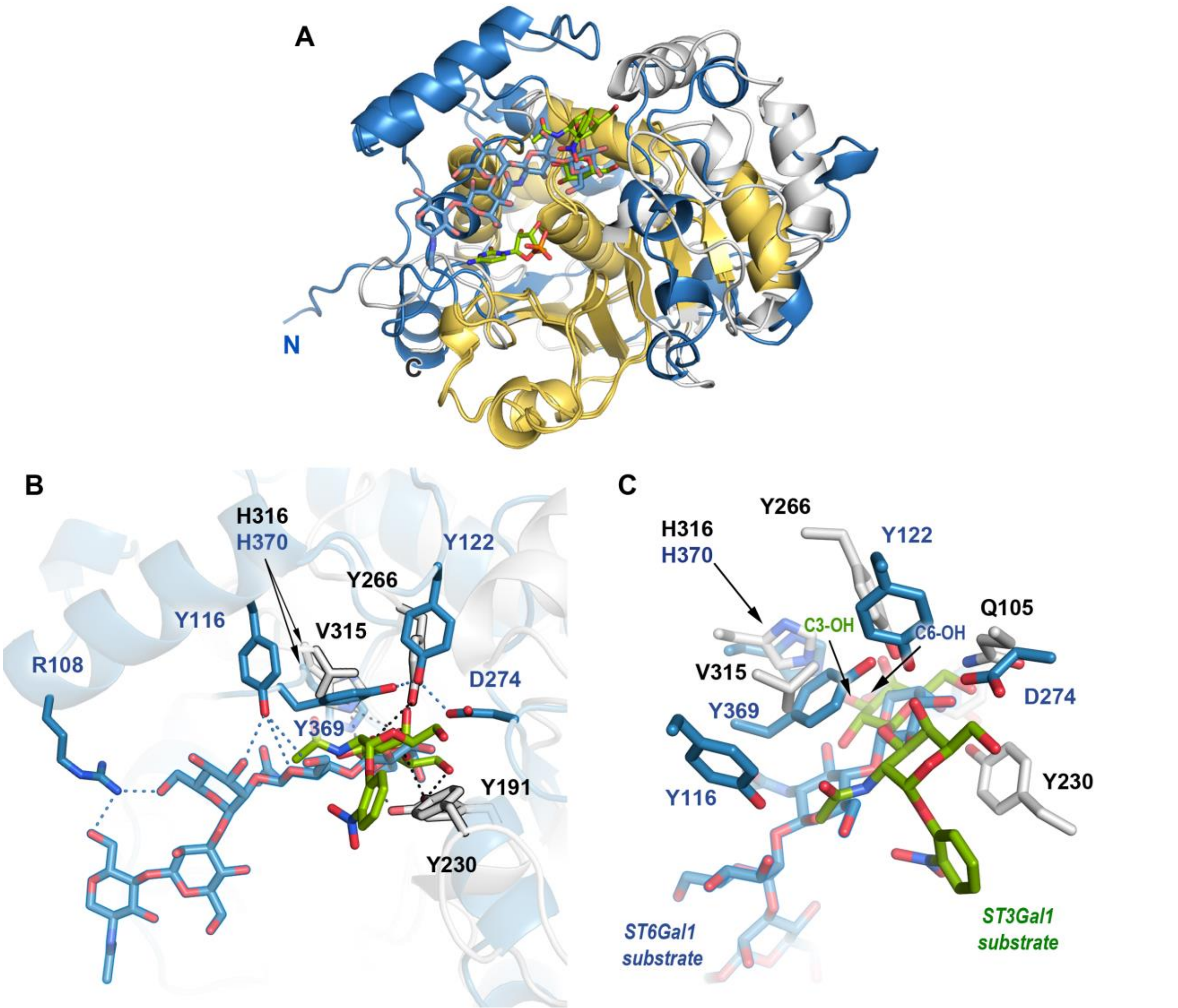

Supplementary Figure S3. Structural comparison of hST3Gal1 (model based on PDB 2wnb, white) and hST6Gal1 (PDB 4js1, blue). A) The conserved structure of both enzymes is displayed in yellow, while structural differences are shown in white and blue following the mentioned colour code. The N- and C-terminus of hST6Gal1 and ST3Gal1, respectively, are labelled. B-C) Substrate binding mode in hST3Gal1 and hST6Gal1. Acceptor Gal-β-1,3-GalNAc-α-$PhNO_2$ (from PDB 2wnb) is shown in green. hST3Gal1 and hST6Gal1 residues are labelled in black and blue font, respectively. Possible contacts (<4 Å) between residues in the binding site and acceptor substrates are shown as dashed lines (colour code as previously mentioned). Residue Y369 is proposed to contribute to the regioselectivity of hST6Gal1 by stabilizing the acceptor terminal galactosyl-moiety via π-π stacking to receive the sialic acid unit in the C6 position. The equivalent position in hST3Gal1 is occupied by V315. C3- and C6-hydroxyl groups of the terminal galactose of ST3Gal1 and ST6Gal1 substrates (in green and blue, respectively) are also labelled.

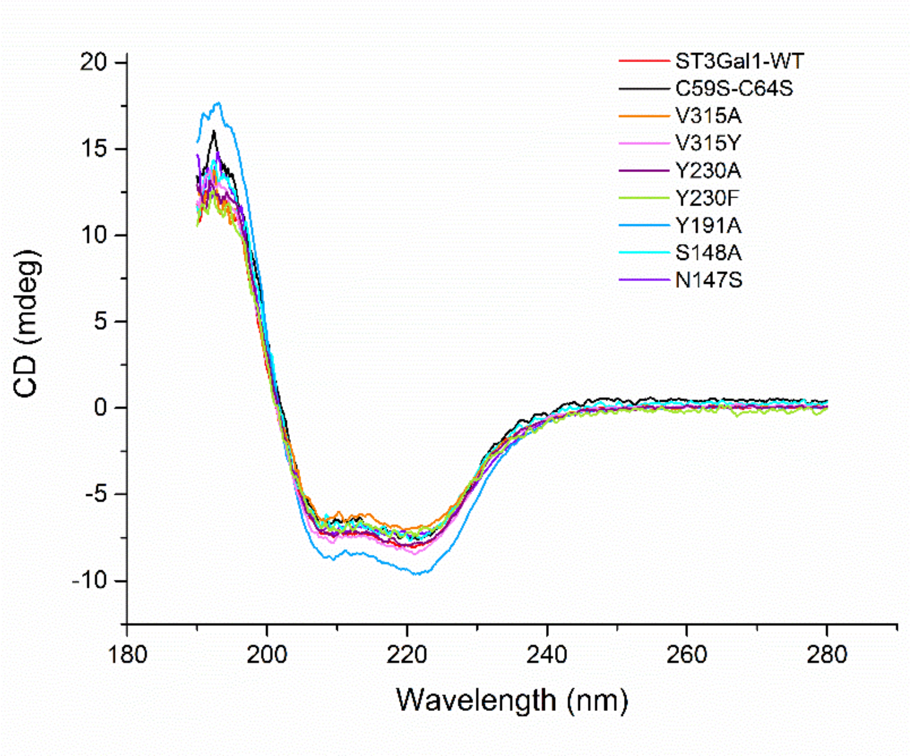

Supplementary Figure S4. Circular dichroism spectra of hST3Gal1 wild-type and variants. CD spectrum of ST3Gal1-WT was previously reported [2].

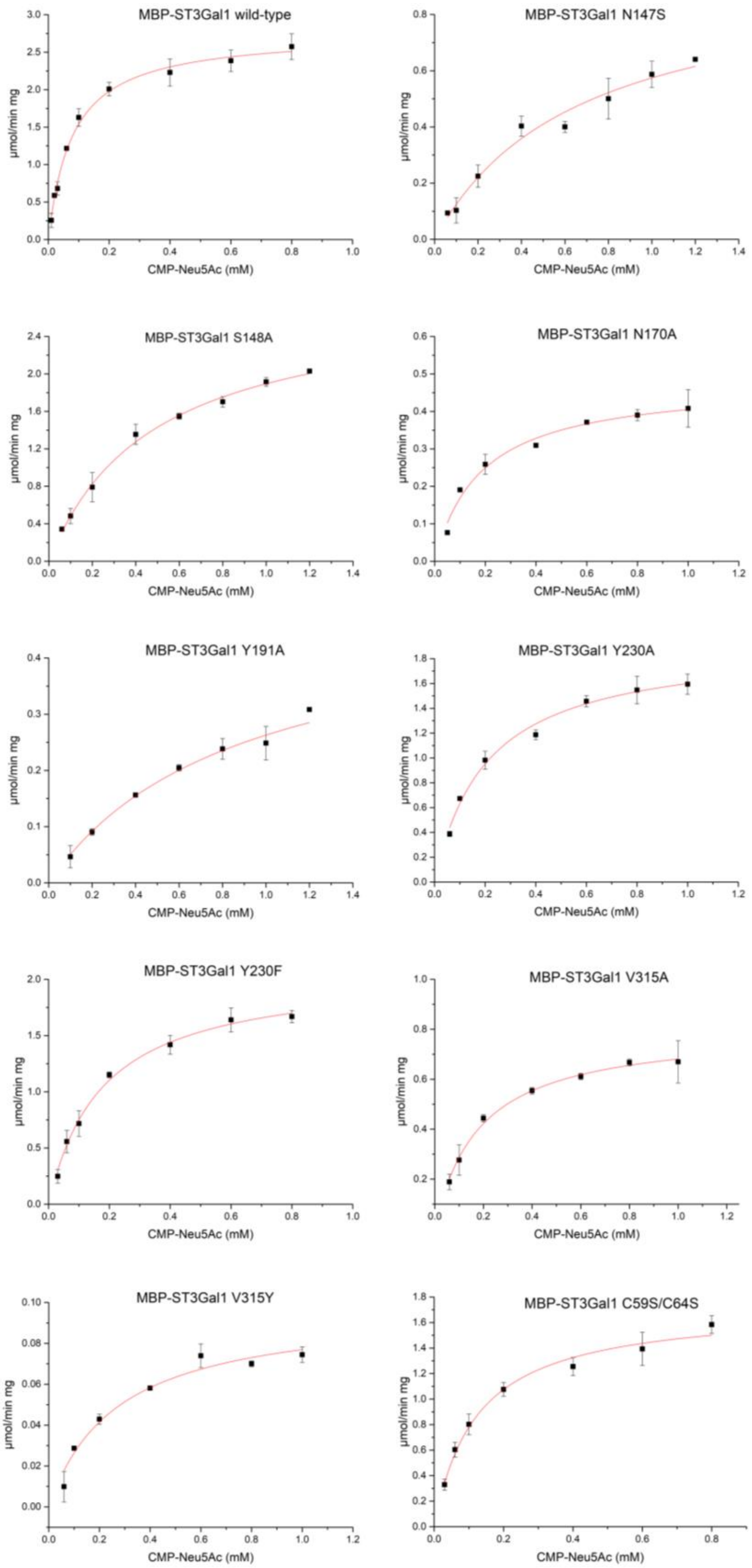

Supplementary Figure S5. Kinetics of MBP-ST3Gal1 wild-type and variants for donor CMP-Neu5Ac. Reactions contained 1 mM Gal-β-1,3-GalNAc-α-*O*-Bn and variable concentrations of donor. Mean values and standard deviations of at least two replicates for each substrate/acceptor concentration are shown. Values for wild-type MBP-ST3Gal1 were previously reported [2].

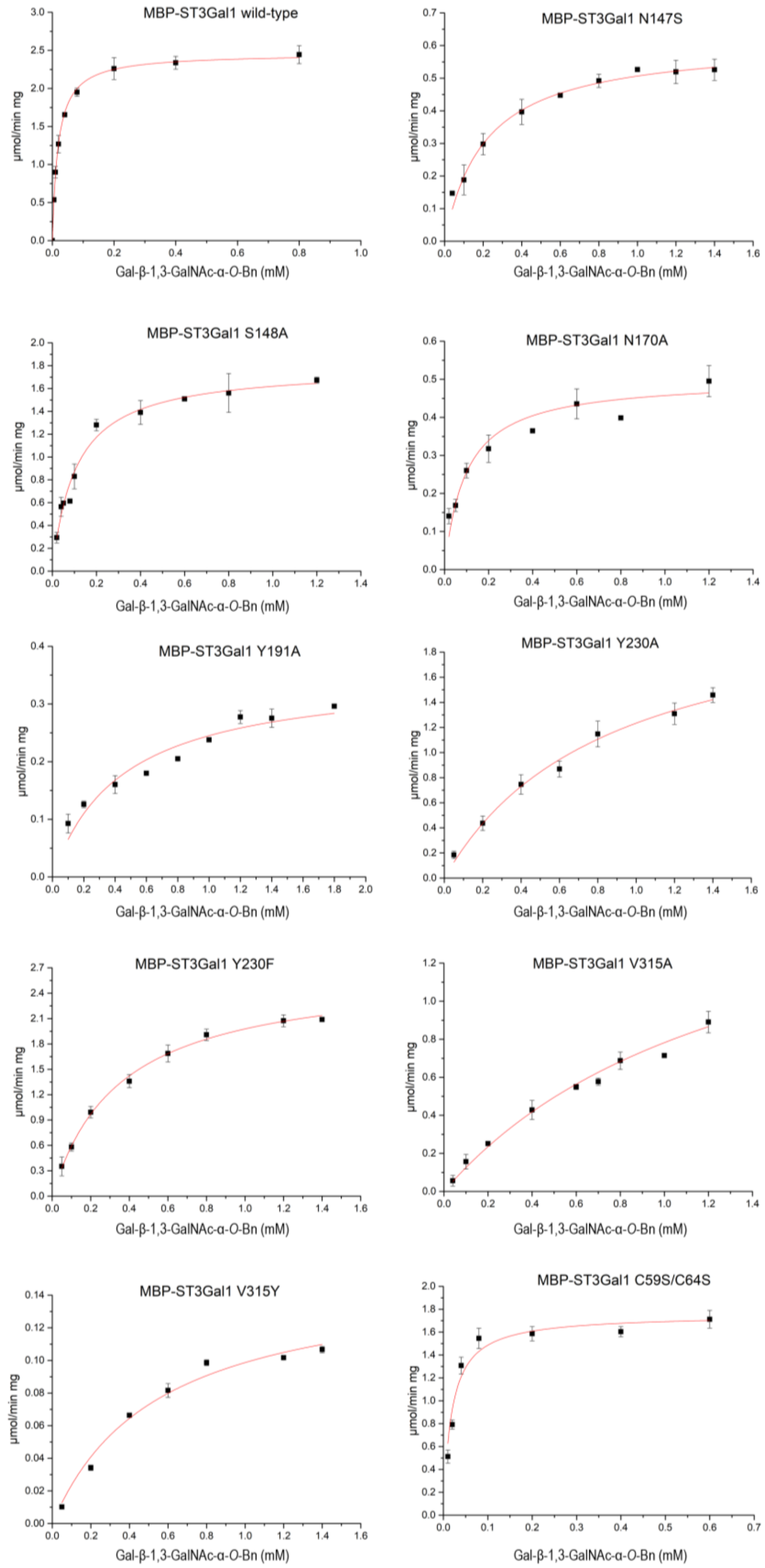

Supplementary Figure S6. Kinetics of MBP-ST3Gal1 wild-type and variants for acceptor Gal-β-1,3-GalNAc-α-*O*-Bn. Reactions contained 0.7 mM CMP-Neu5Ac and variable concentrations of acceptor. Mean values and standard deviations of at least two replicates for each substrate/acceptor concentration are shown. Values for wild-type MBP-ST3Gal1 were previously reported [2].

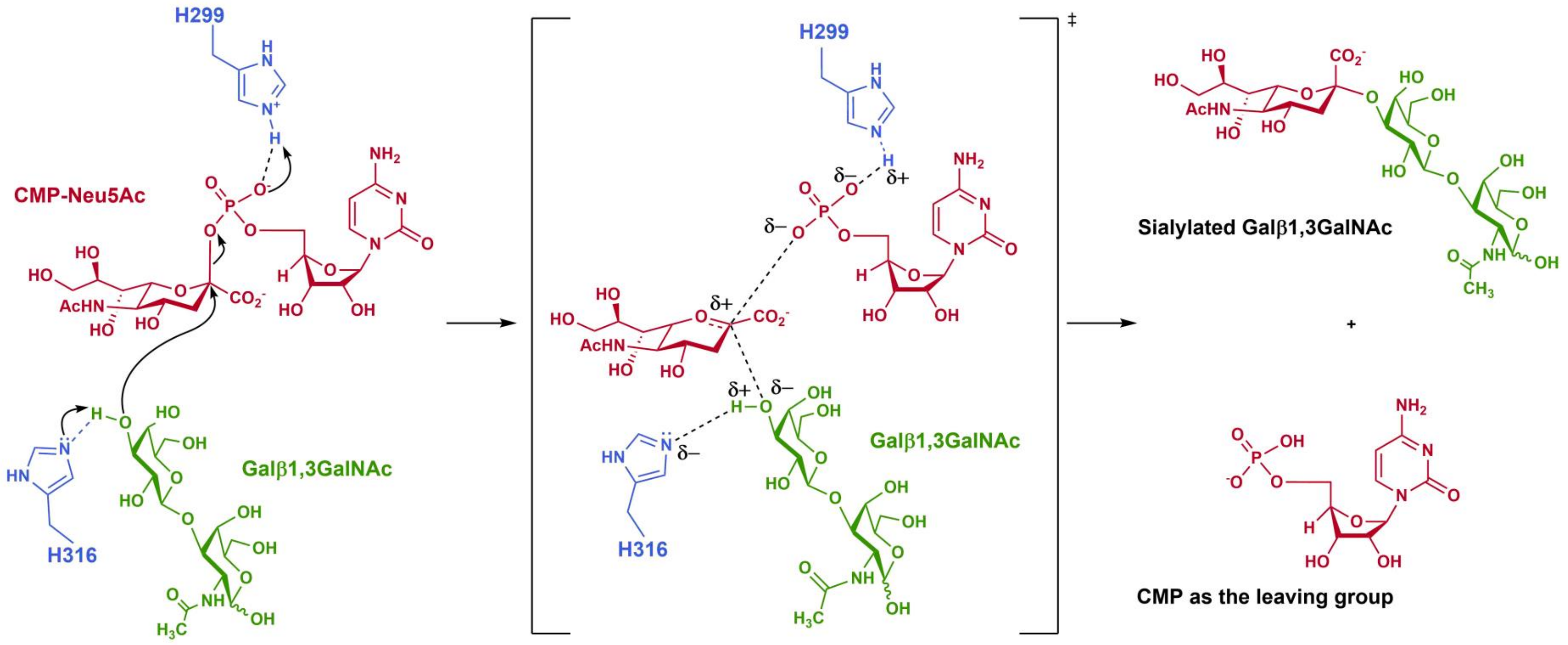

Supplementary Figure S7. Transfer of Neu5Ac onto Gal-β-1,3-GalNAc catalysed by hST3Gal1 proceeding via a S_N2 like mechanism.

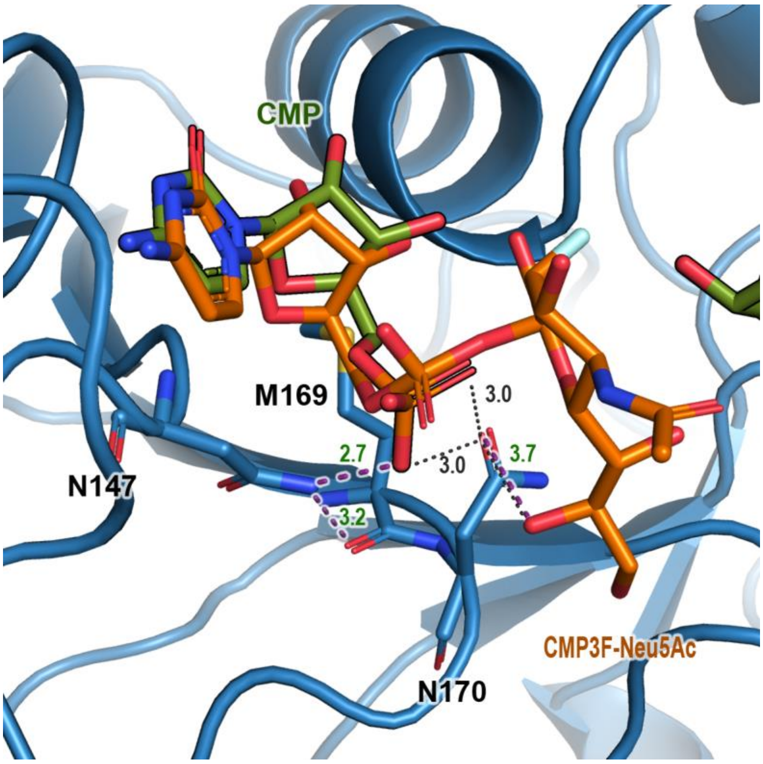

Supplementary Figure S8. Network of possible contacts of hST3Gal1 (model based on PDB 2wnb) residues N147, M169 and N170 with CMP (from PDB 2wnb, green sticks) and CMP3F-Neu5Ac (from the sialyltransferase CstII of *Campylobacter jejuni*, PDB 1ro7, orange sticks). Potential hydrogen bonds (< 4 Å) are shown as dashed lines (purple). The conformation of N170 side chain is transposed in the crystal structures of other sialyltransferases, for example ST6Gal1 (4js2), ST8SiaIII (5bo6) and the sialyltransferase CstII from *Campylobacter jejuni* (1ro7). In those structures asparagine can engage in hydrogen bonds with the donor via the side chain nitrogen. If swapped, N173 could form additional interactions (dotted lines, black) with the phosphate of the leaving group CMP. Structures of CstII and hST3Gal1 were aligned with UCSF Chimera (http://www.rbvi.ucsf.edu/chimera).

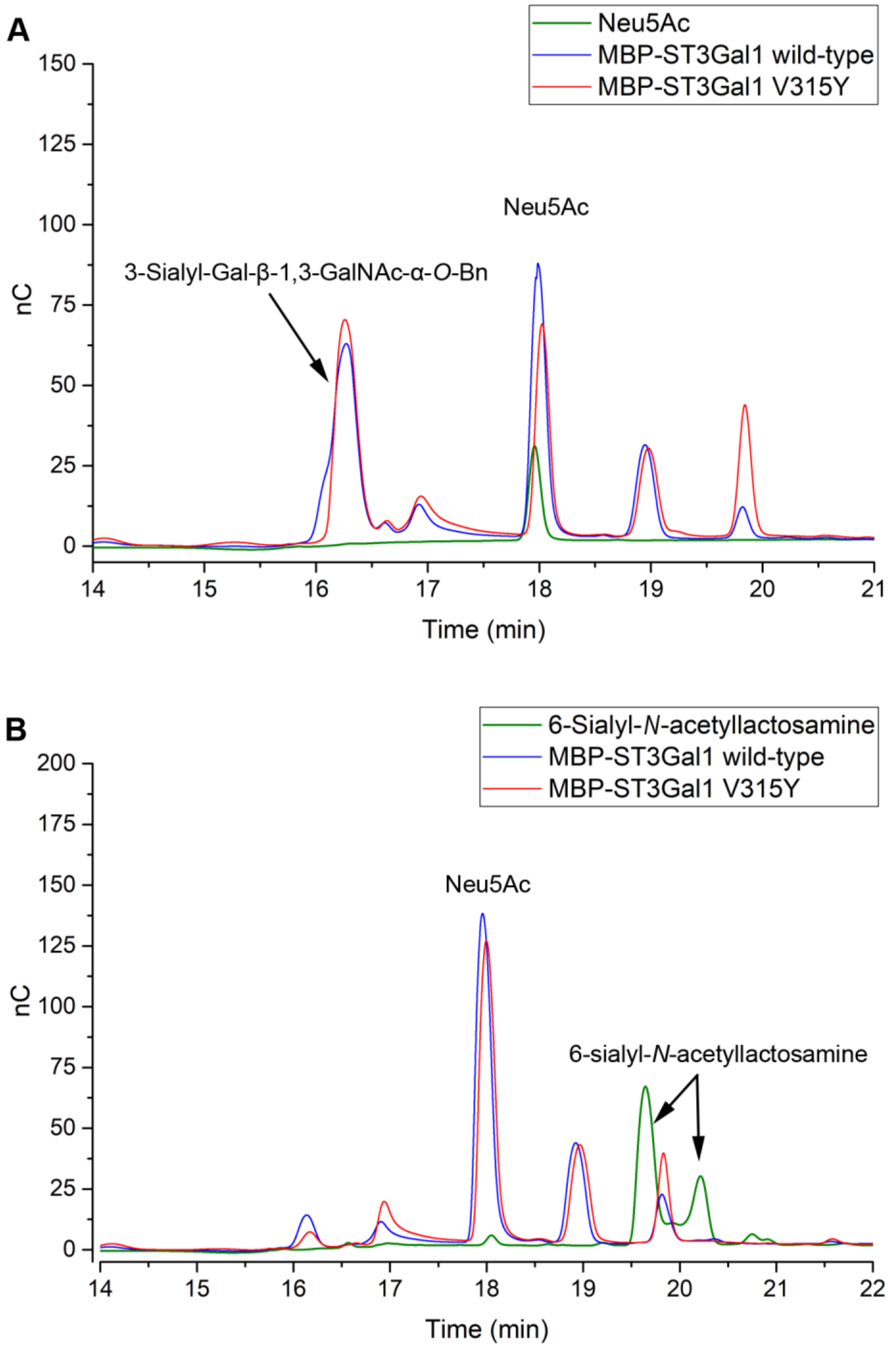

Supplementary Figure S9. Regioselectivity of hST3Gal1 and variant V315Y. HPAEC-PAD product spectra using A) 2 mM Gal-β-1,3-GalNAc-α-*O*-Bn and B) 2 mM LacNAc as acceptor. Reactions were performed during 4 h at 37 °C in 20 mM MOPS pH 7.5 with 3 mM CMP-Neu5Ac as donor and using cleared extracts containing the sialyltransferases. While the yield of 3-sialyl-Gal-β-1,3-GalNAc-α-*O*-Bn was quantitative with hST3Gal1 wild-type and its variant, LacNAc was a rather poor acceptor and the product (3- or 6-sialy-LacNAc) was not detected under the tested reaction conditions. With the elution program employed, the standard 6-sialy-LacNAc appears as a double peak.

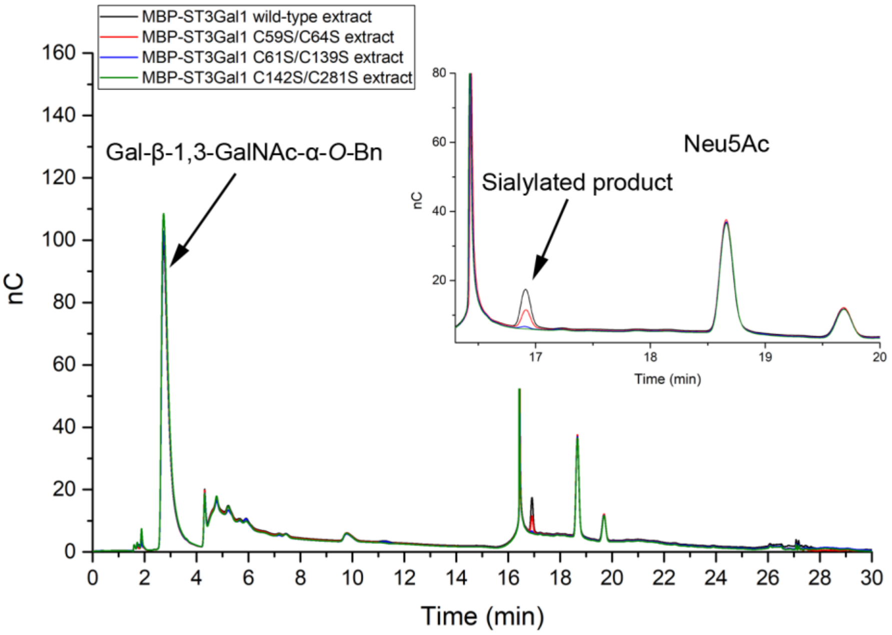

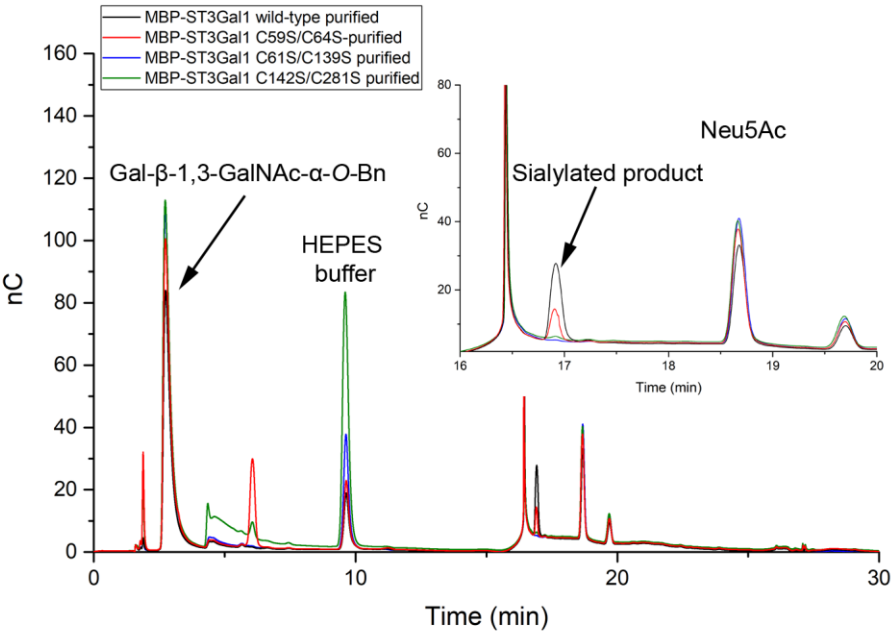

Supplementary Figure S10. HPAEC-PAD product spectra of MBP-ST3Gal1 wild-type and its disulphide variants. Reactions were prepared in 20.0 mM MOPS, pH 7.5 containing either 50 ng μL^{-1} cleared cell lysates with expressed sialyltransferases or 1 ng μL^{-1} purified enzymes (stored in HEPES buffer), 0.5 mM Gal-β-1,3-GalNAc-α-*O*-Bn (acceptor) and 0.7 mM CMP-Neu5Ac (donor). For variant C142S-C281S, a double amount of total (cleared extract) or purified protein was used. Reactions were incubated at 37 °C for 1h.

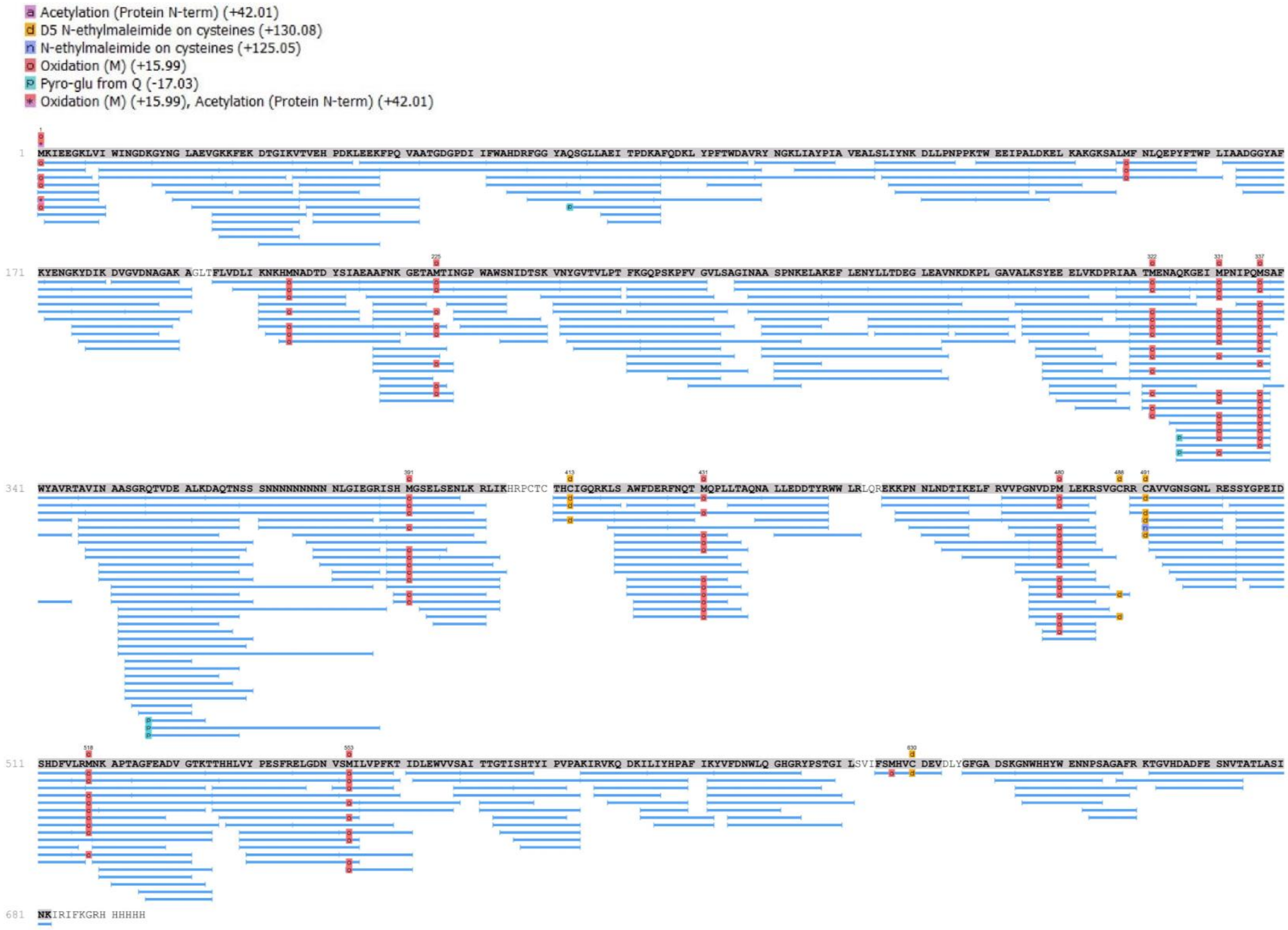
a Acetylation (Protein N-term) (+42.01)
d D5 N-ethylmaleimide on cysteines (+130.08)
n N-ethylmaleimide on cysteines (+125.05)
o Oxidation (M) (+15.99)
p Pyro-glu from Q (-17.03)
* Oxidation (M) (+15.99), Acetylation (Protein N-term) (+42.01)

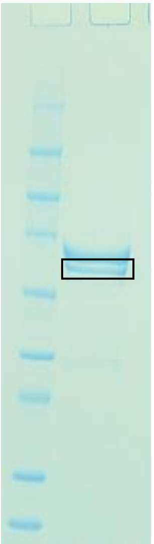

Supplementary Figure S11. Sequence coverage obtained by nanoLC-MS/MS analysis of the protein band below the full length MBP-hST3Gal1 wild-type. Cysteines labelled with *N*-ethylmaleimide (NEM) and NEM-D5 are displayed in purple (n) and orange (d) respectively.

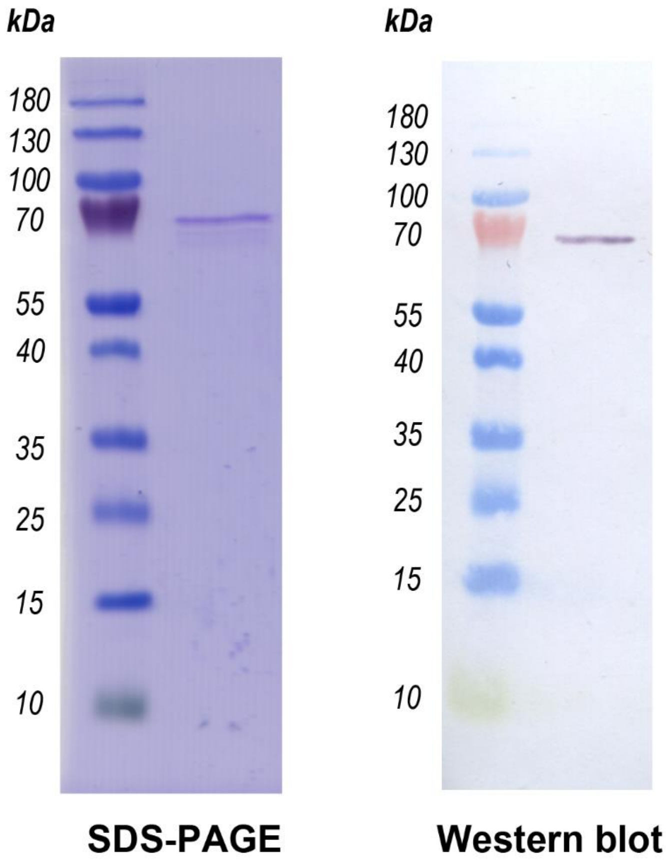

Supplementary Figure S12. SDS-PAGE and western blot of MBP-hST3Gal1 wild-type. The protein was stored at 4 °C for 2 months before performing the SDS-PAGE and blot. The bands observed below the full length protein do not contain the His-tag.

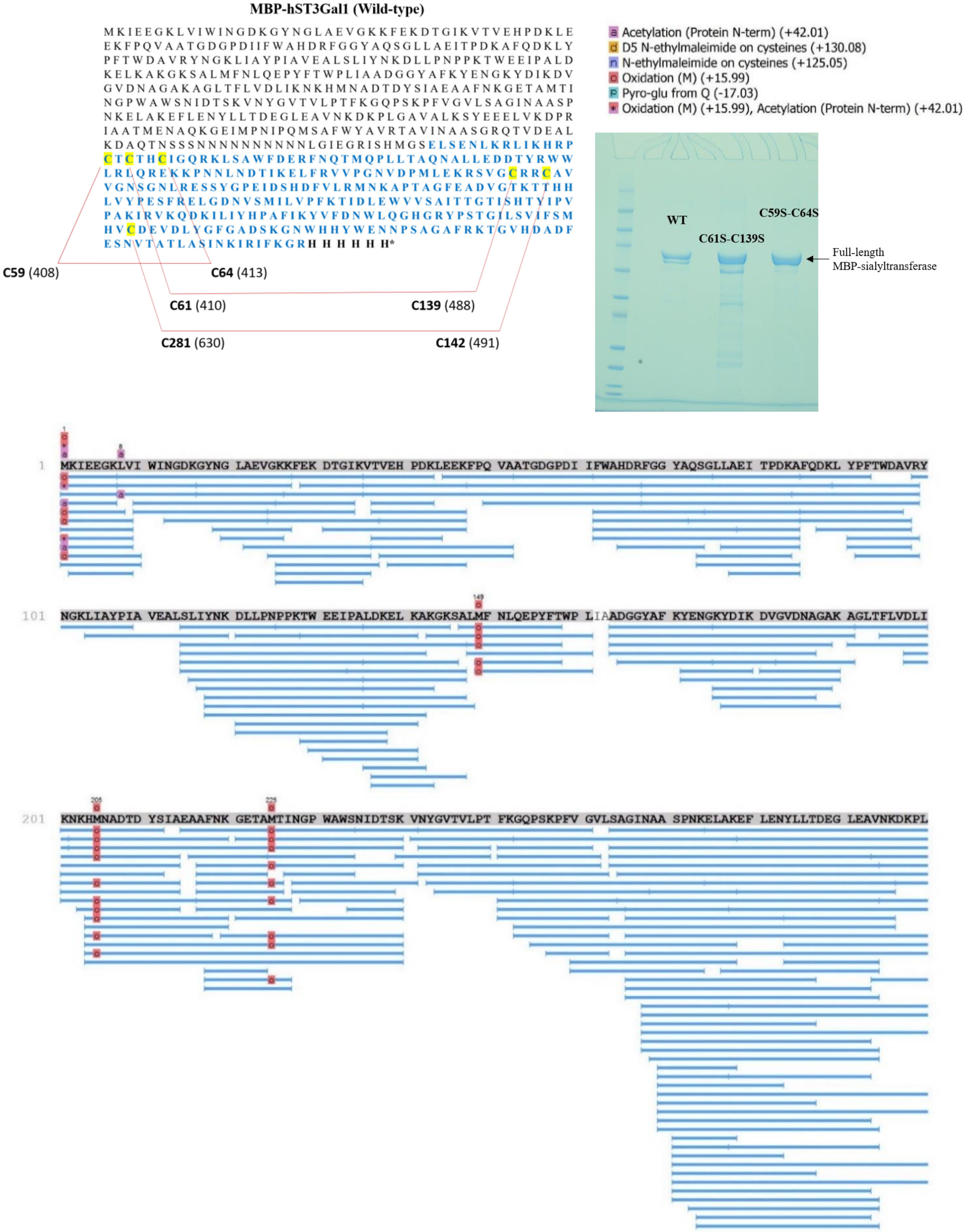

MBP-hST3Gal1 (Wild-type)
C59 (408)
C64 (413)
C61 (410)
C139 (488)
C281 (630)
C142 (491)
Acetylation (Protein N-term) (+42.01)
D5 N-ethylmaleimide on cysteines (+130.08)
N-ethylmaleimide on cysteines (+125.05)
Oxidation (M) (+15.99)
Pyro-glu from Q (-17.03)
Oxidation (M) (+15.99), Acetylation (Protein N-term) (+42.01)
WT
C61S-C139S
C59S-C64S
Full-length
MBP-sialyltransferase

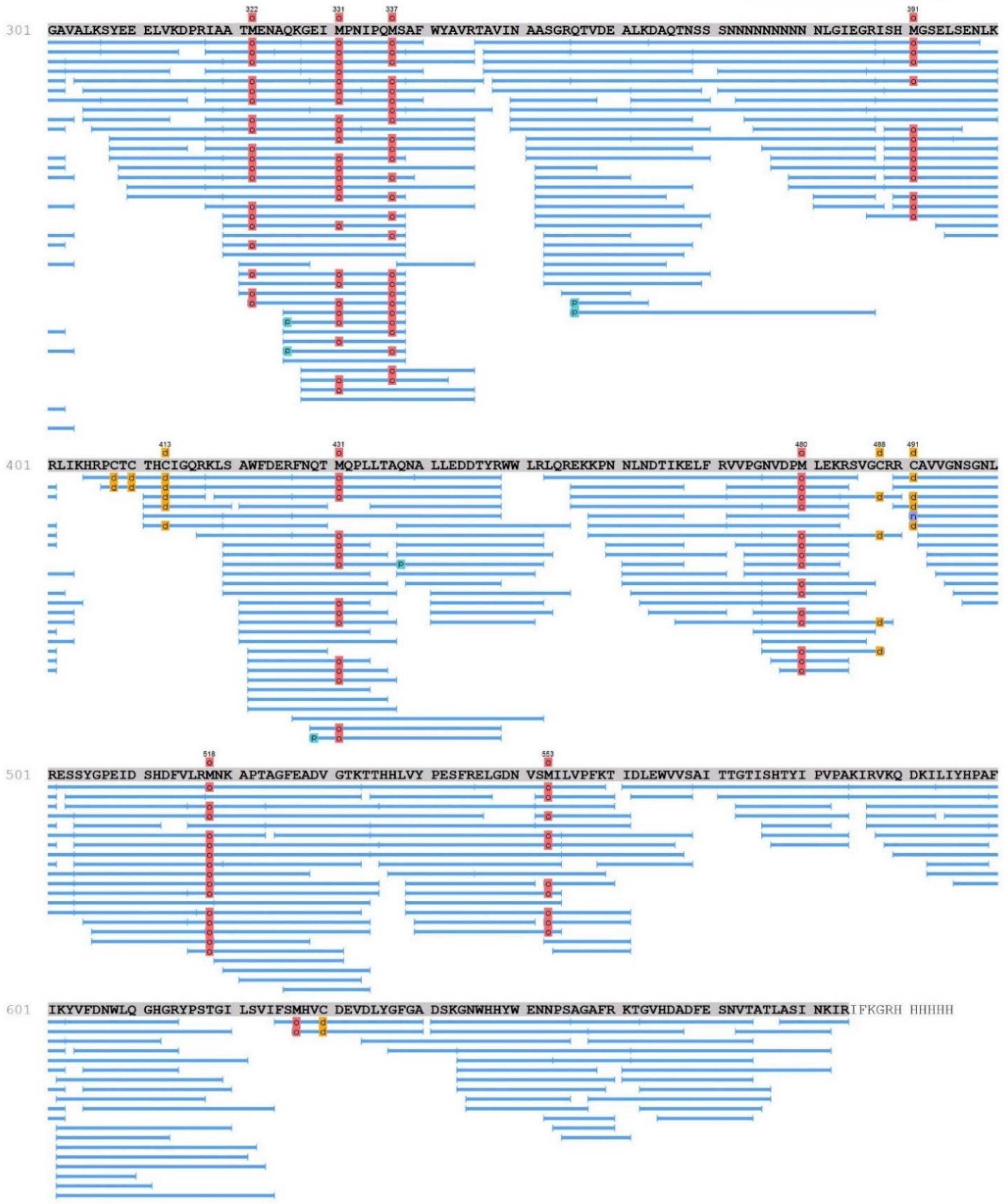

Supplementary Figure S13. Sequence coverage obtained by nanoLC-MS/MS analysis of MBP-hST3Gal1 wild-type. Cysteines labelled with *N*-ethylmaleimide (NEM) and NEM-D5 are displayed in purple (n) and orange (d) respectively.

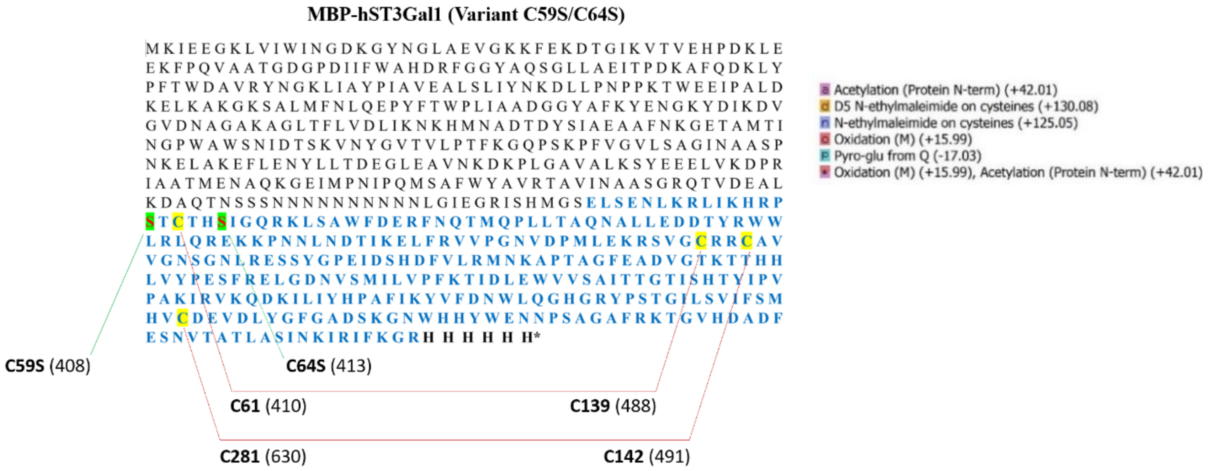
MBP-hST3Gal1 (Variant C59S/C64S)
Acetylation (Protein N-term) (+42.01)
D5 N-ethylmaleimide on cysteines (+130.08)
N-ethylmaleimide on cysteines (+125.05)
Oxidation (M) (+15.99)
Pyro-glu from Q (-17.03)
Oxidation (M) (+15.99), Acetylation (Protein N-term) (+42.01)
C59S (408)
C64S (413)
C61 (410)
C139 (488)
C281 (630)
C142 (491)

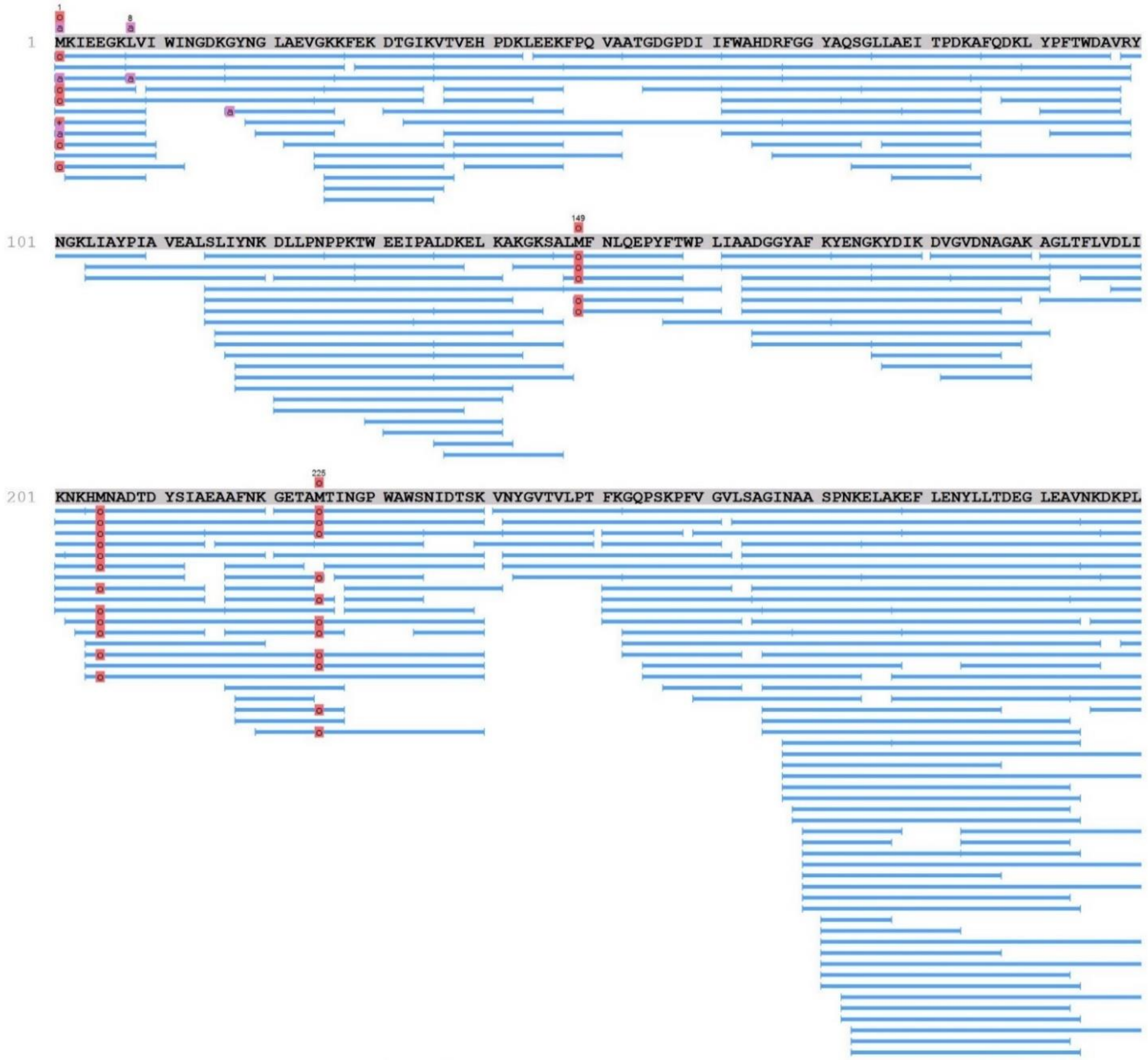

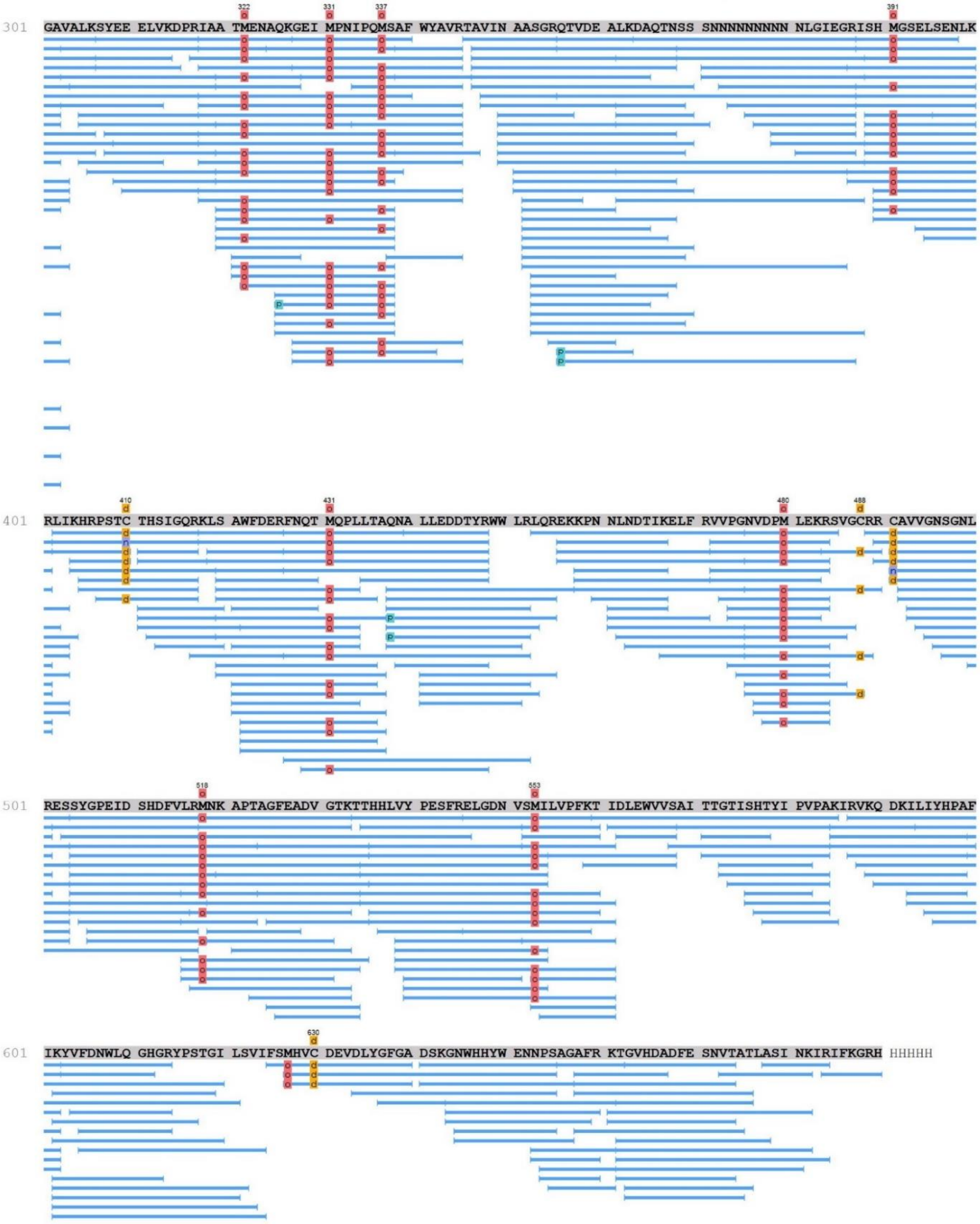

Supplementary Figure S14. Sequence coverage obtained by nanoLC-MS/MS analysis of MBP-hST3Gal1 variant C59S/C64S. Cysteines labelled with *N*-ethylmaleimide (NEM) and NEM-D5 are displayed in purple (n) and orange (d) respectively. Gel band used for the analysis is shown in Supplementary Figure S13.

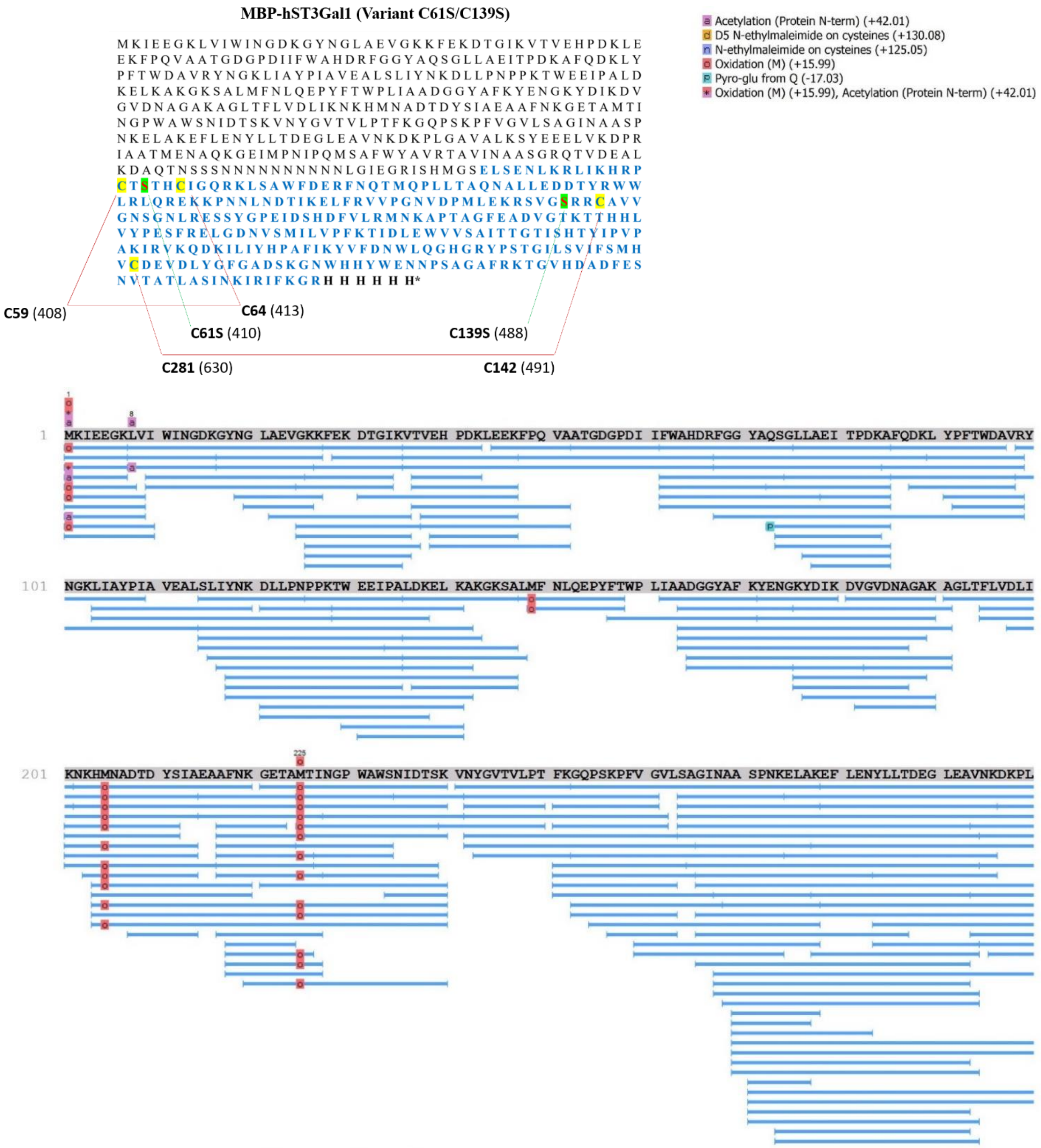
MBP-hST3Gal1 (Variant C61S/C139S)
M K I E E G K L V I W I N G D K G Y N G L A E V G K K F E K D T G I K V T V E H P D K L E
E K F P Q V A A T G D G P D I I F W A H D R F G G Y A Q S G L L A E I T P D K A F Q D K L Y
P F T W D A V R Y N G K L I A Y P I A V E A L S L I Y N K D L L P N P P K T W E E I P A L D
K E L K A K G K S A L M F N L Q E P Y F T W P L I A A D G G Y A F K Y E N G K Y D I K D V
G V D N A G A K A G L T F L V D L I K N K H M N A D T D Y S I A E A A F N K G E T A M T I
N G P W A W S N I D T S K V N Y G V T V L P T F K G Q P S K P F V G V L S A G I N A A S P
N K E L A K E F L E N Y L L T D E G L E A V N K D K P L G A V A L K S Y E E E L V K D P R
I A A T M E N A Q K G E I M P N I P Q M S A F W Y A V R T A V I N A A S G R Q T V D E A L
K D A Q T N S S S N N N N N N N N N N L G I E G R I S H M G S E L S E N L K R L I K H R P
C T S T H C I G Q R K L S A W F D E R F N Q T M Q P L L T A Q N A L L E D D T Y R W W
L R L Q R E K K P N N L N D T I K E L F R V V P G N V D P M L E K R S V G S R R C A V V
G N S G N L R E S S Y G P E I D S H D F V L R M N K A P T A G F E A D V G T K T T H H L
V Y P E S F R E L G D N V S M I L V P F K T I D L E W V V S A I T T G T I S H T Y I P V P
A K I R V K Q D K I L I Y H P A F I K Y V F D N W L Q G H G R Y P S T G I L S V I F S M H
V C D E V D L Y G F G A D S K G N W H H Y W E N N P S A G A F R K T G V H D A D F E S
N V T A T L A S I N K I R I F K G R H H H H H H*
C59 (408)
C64 (413)
C61S (410)
C139S (488)
C281 (630)
C142 (491)
a Acetylation (Protein N-term) (+42.01)
d D5 N-ethylmaleimide on cysteines (+130.08)
n N-ethylmaleimide on cysteines (+125.05)
o Oxidation (M) (+15.99)
p Pyro-glu from Q (-17.03)
* Oxidation (M) (+15.99), Acetylation (Protein N-term) (+42.01)
1
8
1 MKIEEGKLVI WINGDKGYNG LAEVGKKFEK DTGIKVTVEH PDKLEEKFPQ VAATGDGPDI IFWAHDRFGG YAQSGLLAEI TPDKAFQDKL YPFTWDAVRY
101 NGKLIAYPIA VEALSLIYNK DLLPNPPKTW EEIPALDKEL KAKGKSALMF NLQEPYFTWP LIAADGGYAF KYENGKYDIK DVGVDNAGAK AGLTFLVDLI
225
201 KNKHMNADTD YSIAEAAFNK GETAMTINGP WAWSNIDTSK VNYGVTVLPT FKGQPSKPFV GVLSAGINAA SPNKELAKEF LENYLLTDEG LEAVNKDKPL

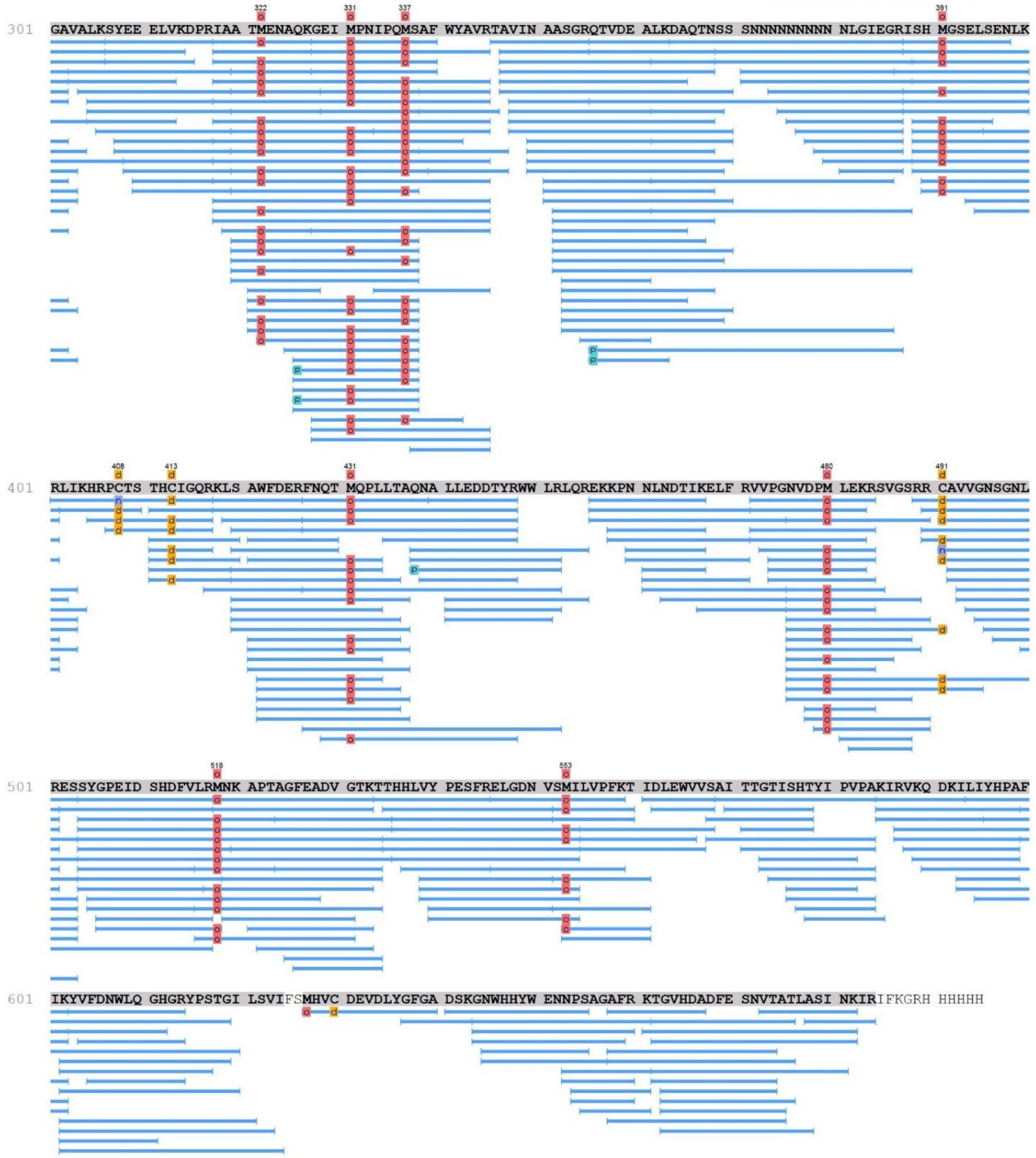

Supplementary Figure S15. Sequence coverage obtained by nanoLC-MS/MS analysis of MBP-hST3Gal1 variant C61S/C139S. Cysteines labelled with *N*-ethylmaleimide (NEM) and NEM-D5 are displayed in purple (n) and orange (d) respectively. Gel band used for the analysis is shown in Supplementary Figure S13.

Supplementary Table S1. The impact of mutations on the transition state stabilization of hST3Gal1 variants.

	**Donor CMP-Neu5Ac**		**Acceptor Galβ1,3GalNAc-α-*O*Bn**	
	**k_{cat}/K_M mM^{-1} min^{-1}**	**$\Delta\Delta G^{\#}$**	**k_{cat}/K_M mM^{-1} min^{-1}**	**$\Delta\Delta G^{\#}$**
**ST3Gal1**[a]	1896	0	7269	0
**Y191A** (Y194)	43	2.3	63	2.9
**Y230A** (233)	732	0.6	208	2.2

The following equation was employed: $\Delta\Delta G^{\#} = 2.303\ RT \log\ (k_{cat}/K_M)_{WT}/(k_{cat}/K_M)_{mutant}$ [3,4]. Where R = 1.987 x 10^{-3} kcal mol^{-1} K^{-1}; T = 310.15 K (37 °C). $\Delta\Delta G^{\#}$ values are rounded.

References

1. Crooks, G. E., Hon, G., Chandonia, J. M. & Brenner, S. E. WebLogo: A sequence logo generator. *Genome Res* **14**, 1188-1190, doi:https://doi.org/10.1101/gr.849004 (2004).
2. Ortiz-Soto, M. E. & Seibel, J. Expression of functional human sialyltransferases ST3Gal1 and ST6Gal1 in *Escherichia coli*. *Plos One* **11**, doi:https://doi.org/10.1371/journal.pone.0155410 (2016).
3. Fersht, A. R. *et al.* Hydrogen-bonding and biological specificity analyzed by protein engineering. *Nature* **314**, 235-238, doi:https://doi.org/10.1038/314235a0 (1985).
4. Rakic, B. *et al.* Structure-based mutagenic analysis of mechanism and substrate specificity in mammalian glycosyltransferases: Porcine ST3Gal-I. *Glycobiology* **23**, 536-545, doi:https://doi.org/10.1093/glycob/cwt001 (2013).
